# m6A modified *BACE1-AS* contributes to liver metastasis and stemness-like properties in colorectal cancer through TUFT1 dependent activation of Wnt signaling

**DOI:** 10.1186/s13046-023-02881-0

**Published:** 2023-11-21

**Authors:** Xidi Wang, Yu Liu, Miao Zhou, Lei Yu, Zizhen Si

**Affiliations:** 1 Central Laboratory of the Medical Research Center, The First Affiliated Hospital of Ningbo University, 247 Renmin Road, Jiangbei District, Ningbo, 315020 P. R. China; 2 Health Science Center, Ningbo University, 818 Fenghua Road, Jiangbei District, Ningbo, 315211 P. R. China; 3 Department of Colorectal Cancer Surgery, The Second Affiliated Hospital of Harbin Medical University, Harbin, P. R. China

**Keywords:** Colorectal cancer, Liver metastasis, m6A modification, *BACE1-AS*, TUFT1

## Abstract

**Background:**

Liver metastasis is one of the most important reasons for high mortality of colorectal cancer (CRC). Growing evidence illustrates that lncRNAs play a critical role in CRC liver metastasis. Here we described a novel function and mechanisms of *BACE1-AS* promoting CRC liver metastasis.

**Methods:**

qRT-PCR and in situ hybridization were performed to examine the *BACE1-AS* level in CRC. IGF2BP2 binding to m6A motifs in *BACE1-AS* was determined by RIP assay and S1m-tagged immunoprecipitation. Transwell assay and liver metastasis mice model experiments were performed to examine the metastasis capabilities of *BACE1-AS* knockout cells. Stemness-like properties was examined by tumor sphere assay and the expression of stemness biomarkers. Microarray data were acquired to analyze the signaling pathways involved in *BACE1-AS* promoting CRC metastasis.

**Results:**

*BACE1-AS* is the most up-regulated in metastatic CRC associated with unfavorable prognosis. Sequence blast revealed two m6A motifs in *BACE1-AS*. IGF2BP2 binding to these two m6A motifs is required for *BACE1-AS* boost in metastatic CRC. m6A modified *BACE1-AS* drives CRC cells migration and invasion and liver metastasis both in vitro and in vivo. Moreover, *BACE1-AS* maintains the stemness-like properties of CRC cells. Mechanically, *BACE1-AS* promoted TUFT1 expression by ceRNA network through miR-214-3p. CRC patients with such ceRNA network suffer poorer prognosis than ceRNA-negative patients. Depletion of TUFT1 mimics *BACE1-AS* loss. *BACE1-AS* activated Wnt signaling pathway in a TUFT1 dependent manner. *BACE1-AS*/miR-214-3p/TUFT1/Wnt signaling regulatory axis is essential for CRC liver metastasis. Pharmacologic inhibition of Wnt signaling pathway repressed liver metastasis and stemness-like features in *BACE1-AS* over-expressed CRC cells.

**Conclusion:**

Our study demonstrated *BACE1-AS* as a novel target of IGF2BP2 through m6A modification. m6A modified *BACE1-AS* promotes CRC liver metastasis through TUFT1 dependent activation of Wnt signaling pathway. Thus, targeting *BACE1-AS* and its downstream Wnt signaling pathways may provide a new opportunity for metastatic CRC intervention and treatment.

**Supplementary Information:**

The online version contains supplementary material available at 10.1186/s13046-023-02881-0.

## Background

Colorectal cancer (CRC) is the third most common malignancy and second leading cause of cancer-related death globally [1]. The major cause of CRC's high mortality is frequent metastasis, which affects 40% of CRC patients, and 90% of metastatic cases die as a result [2]. Liver is one of the most common distant target organs for CRC metastasis [3]. Neoadjuvant therapy and other novel therapeutics for CRC treatment have been developed in recent decades [4]. However, a certain proportion of patients still sustain liver metastasis, calling the emergency of characterizing new factors and underlying molecular mechanisms driving CRC liver metastasis.

Long non-coding RNAs (lncRNAs) are a class of transcripts longer than 200 nucleotides with limited protein-coding potential [5]. Increasing studies have demonstrated dysregulated lncRNA profiles in different cancers, indicating the critical roles of lncRNA in regulating pathological cancer processes [6]. Notably, lncRNAs are involved in all stages of cancer metastasis, from cell migration to distant-organ colonization [7]. For instance, STAT3-mediated lncRNA *HOXD-AS1* up-regulation facilitates hepatocellular carcinoma metastasis through the ceRNA mechanism [8]. LncRNA *VAL* functions as a potent pro-metastatic molecule and is essential for active AKT-induced tumor invasion, metastasis, and anoikis resistance in lung adenocarcinoma [9]. Loss of *XIST* promotes brain colonization of breast cancer by activating MSN-c-Met and reprogramming microglia [10].

Fundamental roles of lncRNAs have been demonstrated in CRC liver metastasis. LncRNA *GAL* promotes colorectal cancer liver metastasis through stabilizing GLUT1 [11]. Elevated *EVADR* serves as a modular scaffold for YBX1 to directly enhance the translation of epithelial-mesenchymal transition (EMT)-related factors and thus promotes CRC liver metastasis. Considering the importance of lncRNAs, further characterization of CRC metastasis-specific lncRNAs and their molecular regulatory mechanisms are promising to provide novel biomarkers and therapeutic targets for CRC liver metastasis.

Beta-secretase 1 antisense RNA (*BACE1-AS*) is a lncRNA transcribed from the opposite strand of β-secretase 1 (*BACE1*). Previous studies reported an important role of *BACE1-AS* together with *BACE1* in Alzheimer's disease [12]. Recently, increasing evidences demonstrate that BACE1-AS regulates cancer biological processes. BACE1-AS promotes hepatocellular carcinoma progression via miR-377-3p/CELF1 axis [13]. BACE1-AS is also found as an immune-related influencing factor in tumorigenesis [14]. However, the role of BACE1-AS in CRC is unknown.

Tuftelin 1 (TUFT1) is characterized as a key regulator in developing and mineralizing tooth tissues [15], and also recognized as a pluripotency-related gene [16, 17]. TUFT1 is also increased and is associated with poor prognosis in multiple cancers [18]. Although a few studies reported that TUFT1 promotes CRC progression through PI3K/AKT pathway and AKT/GSK-3β/p65 axis [19, 20], the mechanism by which up-regulates TUFT1 in CRC and the role of TUFT1 in CRC have not been discovered.

Our current study found a series of lncRNAs dysregulated in metastatic CRC by microarray dataset and identified beta-secretase 1 antisense RNA (*BACE1-AS*) as the most highly elevated lncRNA with poor prognosis. N6-methyladenosine (m6A) modification was proved to be critical for regulating *BACE1-AS* level. IGF2BP2 recognized and stabilized m6A-modified *BACE1-AS* in metastatic CRC. Functional studies revealed that *BACE1-AS* promoted liver metastatic ability and stemness-like properties of CRC cells both in vitro and in vivo. Mechanistic experiments elucidated that *BACE1-AS* enhanced TUFT1 dependent Wnt signaling pathway activation through competing binding to miR-214-3p. Enforced expression of TUFT1 rescued *BACE1-AS* loss-induced declines of liver metastasis and stemness features of CRC cells. Pharmacologic inhibition of Wnt signaling pathway repressed liver metastasis and stemness-like features in *BACE1-AS* over-expressed CRC cells. Our findings revealed a reasonable cause for *BACE1-AS* up-regulation in CRC liver metastasis and the important regulatory axis of *BACE1-AS*/TUFT1/Wnt signaling pathway, implying that *BACE1-AS* could serve as a potential prognostic marker and possible determination of therapeutic modality for metastatic CRC treatment.

## Materials and methods

### Clinical tissue collection

Four human non-metastatic CRC tissues and four liver metastatic CRC were obtained from the Second Affiliated Hospital of Harbin Medical University with the patient's consent. The research method was approved by the Ethics Committee of Harbin Medical University (KY2016-036). All CRC tissues were immediately frozen and preserved in liquid nitrogen until use.

### Cell culture

CCD841 CoN human normal colonic epithelial cell line was obtained from American Type Culture Collection (ATCC, Manassas, VA, USA) and cultured in Eagle's Minimum Essential Medium (EMEM, Lonza, Basel, Switzerland), supplemented with 10% FBS. In addition, four human CRC cell lines, including SW480, HCT116, SW620, and LoVo cells, were purchased from the Cell Bank of the Chinese Academy of Sciences (Shanghai, China). All CRC cell lines were cultured in Dulbecco's modified Eagle medium (Invitrogen, Carlsbad, CA, USA), supplemented with 10% fetal bovine serum (FBS; Invitrogen), 50 U/mL penicillin, and 50 μg/mL streptomycin (Invitrogen). All cells were maintained in a humidified incubator at 37℃ with 5% CO2.

### Lentivirus infection

Lentiviruses carrying specific shRNA targeting *BACE1-AS* or *BACE1-AS* over-expression elements were purchased from GeneCopoeia, Guangzhou, China. For lentivirus infection, HCT116 cells or SW620 cells were seeded into 60 mm dishes 24 h before infection. After adherence, indicated lentivirus was added to cells, followed by 48 h of culture. Then, cells were lysed for further experiments.

### Construction of m6A motif mutant *BACE1-AS*

The wild-type *BACE1-AS* expression vector was purchased from GeneCopoeia, Guangzhou, China. Then, a point mutation was introduced into *BACE1-AS* to generate single or double m6A motifs mutant *BACE1-AS* using Phusion Site-Directed Mutagenesis Kit (Thermo Fisher Scientific, Waltham, MA, USA), and named as *BACE1-AS*-M1 (motif 1 single mutant) or *BACE1-AS*-M2 (motif 2 single mutant) or *BACE1-AS*-M1/M2 double mutant). Finally, the correct mutant *BACE1-AS* was examined by DNA sequencing.

### Generation of *BACE1-AS* knock-out SW620 cells

The *BACE1-AS* knockout SW620 cells was customized in Cyagen Biosciences, Suzhou, China. The only exon of *BACE1-AS* was completely deleted to generate *BACE1-AS* knockout SW620 cells. The knockout clones were identified by PCR using genome DNA as a template. qRT-PCR was performed to verify the *BACE1-AS* expression in knockout cells.

### RNA isolation and qRT-PCR

Total RNA was isolated using TRIzol reagent (Invitrogen) according to manufacturer protocol. First, the RNA concentrations were measured by a Nanodrop spectrophotometer (Thermo Fisher Scientific). Next, 1 μg total RNA was transcribed into cDNA by high-capacity cDNA reverse transcription kit (Thermo Fisher). Then, qRT-PCR was performed using Power SYBR Green PCR master Mix (Thermo Fisher Scientific). The PCR conditions were 30 cycles at 94 °C for 20 s, 60 °C for 20 s, and 72 °C for 30 s. Relative gene expression levels were calculated by the 2-ΔΔCt method. The primers for examining *BACE1-AS* expression are listed as follows:

- *BACE1-AS*-F: 5’- TGGCTGTTGCTGAAGAATGTGACTC -3’;
- *BACE1-AS*-R: 5'- CAACCTTCGTTTGCCCAAGAAAGTG -3'
- WNT3A-F: 5’- TCTTACTCCTCTGCAGCCTGA -3’;
- WNT3A-R: 5’- GTTCCTGCAGAAGCGGAGCTG -3’;
- WNT7B-F: 5’- CACAGAAACTTTCGCAAGTGG -3’;
- WNT7B-R: 5’- GTACTGGCACTCGTTGATGC -3’;
- CTNNB1-F: 5’- CATCTACACAGTTTGATGCTGCT -3’;
- CTNNB1-R: 5’- GCAGTTTTGTCAGTTCAGGGA -3’;
- MYC-F: 5’- GTCAAGAGGCGAACACACAAC -3’;
- MYC-R: 5’- TTGGACGGACAGGATGTATGC -3’;
- SOX2-F: 5’- GCCGAGTGGAAACTTTTGTCG -3’;
- SOX2-R: 5’- GGCAGCGTGTACTTATCCTTCT -3’;
- FOSL1-F: 5’- CAGGCGGAGACTGACAAACTG -3’;
- FOSL1-R: 5’- TCCTTCCGGGATTTTGCAGAT -3’;
- β-actin-F: 5’- CCTGGACTTCGAGCAAGAGATGG -3’;
- β-actin-R: 5’- CAGGAAGGAAGGCTGGAAGAGTG -3’.
- β-actin was used for normalization.

In order to examine the expression of miR-214-3p, 1 μg of total RNA was transcribed into cDNA using miR-214-3p specific stem-loop primer. The condition of qRT-PCR to detect miR-214-3p was the same as described above using a miR-214-3p specific primer set.

miR-214-3p specific stem loop primer:

- GTCGTATCCAGTGCGTGTCGTGGAGTCGGCAATTGCACTGGATACGACACTGCCTG
- miR-214-3p-F: 5’- GGGACAGCAGGCACAGACAG -3’.
- miR-214-3p-R: 5'- CGTATCCAGTGCGTGTCGTG -3'.
- U6-F: 5’- GCTTCGGCAGCACATATACTAAAAT -3’
- U6-R: 5'- CGCTTCACGAATTTGCGTGTCAT -3'
- U6 was used for normalization.

All primer sequences were synthesized from Sangon Biotech, Shanghai, China.

### Immunoblotting

Treated cells were lysed using RIPA lysis buffer supplemented with a protease inhibitor cocktail (Roche, Switzerland). The protein concentrations of each sample were measured by Quick Start™ Bradford 1 × Dye Reagent (Bio-Rad, Hercules, CA, USA). Equal proteins were separated by SDS-PAGE and transferred to nitrocellulose membranes (PALL, MA, USA). Then, the membranes were blocked with 5% skim milk, and the blots were probed with primary antibodies against IGF2BP2 (ab129071), TUFT1 (ab184949), NANOG (ab109250) (Abcam, Cambridge, MA, USA), OCT4 (#2750), FOSL1 (#5281) (Cell Signaling Technology, Danvers, MA, USA), β-catenin (51,067–2-AP), c-Myc (10,828–1-AP), SOX2 (11,064–1-AP) (Proteintech, Wuhan, China) and Actin (Santa Cruz Biotechnology, Santa Cruz, CA, USA) overnight in 4 °C. After washing with PBST, the membranes were incubated with anti-rabbit (#7074) or anti-mouse (#7076) secondary antibodies (Cell signaling Technology), and the blots were visualized by ECL reagent (GE Healthcare, USA).

### In situ hybridization (ISH)

The ISH assay was performed to examine the level of *BACE1-AS* in paraffin-embedded CRC tissues and adjacent normal tissues collected from CRC patients. In brief, after deparaffinization and rehydration, the slices were permeabilized with proteinase K. Later, the slices were hybridized with digoxigenin (DIG) labeled anti-*BACE1-AS* probe over-night. Next day, the slices were washed for 15 min followed by incubation with anti-DIG horseradish peroxidase-conjugated secondary antibody (SE266, SolarBio Technology, Beijing, China) for 1 h at room temperature. Then, the slices were subjected to DAB staining and photographed using an Olympus BX51 microscope. All images were evaluated by two pathologists.

### RNA binding protein immunoprecipitation (RIP) assay

The m6A enrichment in *BACE1-AS* was measured by RIP assay using an m6A antibody (68,055–1-Ig, Proteintech). In brief, total RNAs were isolated from SW620 cells or non-metastatic or metastatic CRC tissue samples and dissolved in 50 μl sterilized RNase-free water. Thereinto, 5 μl solution was saved as input fraction, and the rest 45 μl was added into RIP buffer supplemented with RNase inhibitor. The RNA samples were pre-cleaned with mouse IgG (#37,988, Cell Signaling Technology). Then, pre-cleaned samples were incubated with mouse IgG or m6A antibody overnight at 4 °C, followed by incubation with protein A/G beads at 4 °C. Then, the immunoprecipitated m6A-modified RNAs were harvested. The RNA concentrations were measured using a NanoDrop spectrophotometer. Purified RNA was reverse transcribed into cDNA using random primers and subjected to qPCR to examine the level of m6A-modified *BACE1-AS*.

The interaction between *BACE1-AS* and IGF2BP2 was also examined by RIP assay using the IGF2BP2 antibody (Abcam) using the same protocol above. In addition, the level of *BACE1-AS* in IGF2BP2 antibody immunoprecipitated pellets was examined by qRT-PCR using a specific primer set for *BACE1-AS*.

### S1m-tagged *BACE1-AS* immunoprecipitation

pCDH-MSCV-4xS1m-GFP-T2A-PU vector carrying S1m tag was obtained from Addgene [21]. As a template, the *BACE1-AS* sequence was amplified by PCR using cDNA samples from HEK293 cells. Later, the *BACE1-AS* sequence was inserted into the pCDH-MSCV-4xS1m-GFP-T2A-PU vector, and the correct clone was examined by DNA sequencing and named S1m-*BACE1-AS*.

In order to pull down S1m-tagged *BACE1-AS*, SW620 cells transfected with the S1m-*BACE1-AS* vector were lysed, and the supernatant was collected by centrifugation. Then, cell lysate was incubated with MagnaBind™ streptavidin beads (Thermo Fisher Scientific) for 1 h at 4 °C to eliminate non-specific binding. Next, the pre-cleaned lysate was transferred to a new tube, and 50 μl was saved as input. Next, the rest solution was incubated with streptavidin beads overnight at 4 °C followed by 4 times washing with pre-cold PBS. Finally, the immunoprecipitated pellet was dissolved in 30 µl 2 × protein loading buffer and examined by western blotting using IGF2BP2 antibody.

### Wound healing assay

SW620 cells infected with lentivirus carrying scramble sequence or shRNA sequence targeting *BACE1-AS* were seeded in 35 mm dishes and grown to a 70—80% density. Then, an artificial wound was created by scratching using a 200-μl pipette tip. Images were captured at 0 and 48 h after scratching, and the migration speed was calculated.

### Trans-well assay

For migration analysis, SW620 cells infected with lentivirus carrying scramble sequence or shRNA sequence targeting *BACE1-AS* were seeded in the upper chamber of a 24-well trans-well unit (5 × 10^4^ cells/well) with 8-μm polycarbonate nucleopore filters (Corning Costar, Corning, NY, USA). The upper compartment contained serum-free medium, while the lower compartment contained medium supplied with 10% FBS. Cells were then incubated under 37 °C and 5% CO2 in a humid incubator. Crystal violet staining was used 24 h later to fix and count the cells adhering to the lower surface of the filter.

For the invasion assay, the membrane of the 24-well trans-well unit was coated with 40μL Matrigel (BD Biosciences, San Jose, CA, USA) and incubated at 37 °C for 4 h to form a reconstructed basement membrane. The invasion abilities of cells were examined using the same protocol as that used for the migration assay.

### Tumor sphere formation assay

In total, 2 × 10^3^ SW620 cells transfected with indicated reagents or *BACE1-AS* knockout or parental SW620 cells were seeded into 6-well plates, and cultured with FBS-free DMEM/F-12 (1:1) medium (Gibco, Grand Island, NY, USA) supplemented with 10 ng/ml epidermal growth factor, 10 ng/ml basic fibroblast growth factor and N-2 supplement (Gibco) on ultra-low attachment six-well plates (Corning). After 2-week culture, the formed tumor spheres were photographed and counted. Meanwhile, the diameters of the formed spheres were measured.

### In vivo liver metastasis formation experiment

Immune-compromised NOD SCID mice (6-week-old) were obtained from Vital River Laboratory, Beijing, China. All mice were housed under specific pathogen-free (SPF) conditions and handled using aseptic procedures. Then, the mice were randomly assigned to two groups for further experiments.

*BACE1-AS* knockout SW620 or parental SW620 cells were injected into the inferior Hemi-spleen of the mice. The weights of mice were recorded every 3 days. After 7 weeks, the mice were euthanized. The whole livers of mice were resected and photographed to assess metastatic burden.

### Dual-luciferase reporter assay

We used Starbase to predict the binding sites of miR-214-3p in *BACE1-AS* and TUFT1. Sequences containing the predicted binding site and their flanking sequences (~ 200 bp) were amplified using RNA samples from HEK293 cells as a template and cloned into the SpeI and HindIII sites of the pMIR-REPORT Luciferase vector (Ambion, Austin, TX, USA), and named as *BACE1-AS*-WT and TUFT1-WT. Several point mutations were introduced into *BACE1-AS*-WT and TUFT1-WT using Phusion Site-Directed Mutagenesis Kit to generate a binding site mutant vector and named *BACE1-AS*-Mut and TUFT1-Mut.

SW620 cells were seeded into 6-well plates and transfected with indicated vectors using Lipofectamine 3000 (Invitrogen) for 48 h to see if miR-214-3p directly targeted *BACE1-AS* and TUFT1. Later, luciferase activities in each group were examined using the Dual-luciferase reporter 1000 assay system (Promega, Madison, WI, USA). Renilla luciferase activity was used for normalization.

### Data analysis

Data were obtained from at least 3 biological individual experiments and presented as the mean ± standard deviation. Statistical data were analyzed using Statistical Program for Social Sciences (SPSS) 17.0 software (SPSS, Chicago, IL, USA). Two-tailed Student's t-test analyzed the comparison between two groups. ANOVA analyzed comparisons among three groups or more than three groups. *P* < 0.05 was considered to represent a significant difference. *p < 0.05, **p < 0.01, ***p < 0.001.

## Results

### Elevated *BACE1-AS* in metastatic CRC is stabilized by m6A modification

**Fig. 1:**
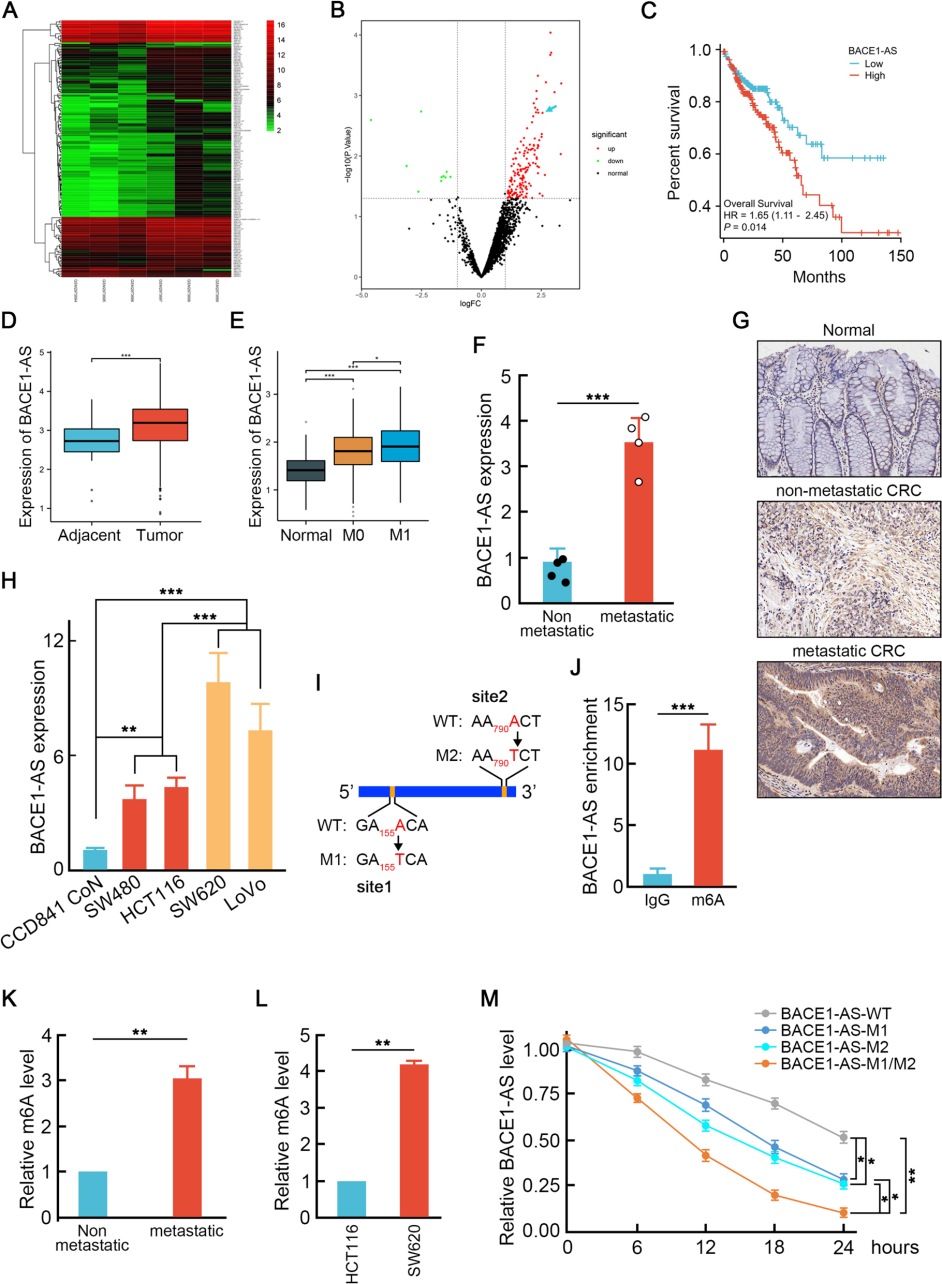
m6A modification promotes *BACE1-AS* elevation in metastatic CRC. **A** and **B** The heatmap (**A**) and a volcano plot (**B**) of differentially expressed lncRNAs (DELs) between metastatic and non-metastatic CRC tissue samples. Non-differentially expressed lncRNAs (gray), up-regulated lncRNAs (red), and down-regulated lncRNAs (green) were shown in different colors (**B**). Blue arrow indicated *BACE1-AS*. **C** Higher *BACE1-AS* indicated poorer prognosis of CRC patients. **D** *BACE1-AS* was higher in CRC tissues compared to adjacent normal tissues. Data from TCGA database. ***p < 0.001. **E** *BACE1-AS* was increased in metastatic CRC compared to normal tissues or non-metastatic CRC. *p < 0.05, ***p < 0.001. **F** Higher *BACE1-AS* was found in metastatic CRC tissues than in non-metastatic CRC tissues. ***p < 0.001. **G** In situ hybridization assay was performed to assess the *BACE1-AS* levels in adjacent normal tissues, non-metastatic and metastatic CRC tissues. **H** *BACE1-AS* expression in CCD841 CoN normal human colonic epithelial cell line as well as in low (SW480 and HCT116) or high (SW620 and LoVo) metastasis potential CRC cell lines were examined by qRT-PCR. **p < 0.01, ***p < 0.001. **I** Schematic diagram of two potential m6A motifs in *BACE1-AS*. **J** Enrichments of *BACE1-AS* in m6A antibody precipitated pellets. **K** Higher m6A level was found in metastatic CRC tissues than in non-metastatic CRC tissues. **L** Higher m6A level was found in high metastasis potential SW620 cells than in low metastasis potential HCT116 cells. **M** Mutations of m6A motifs in *BACE1-AS* facilitated *BACE1-AS* decay. *p < 0.05, **p < 0.01

To uncover the dysregulated lncRNAs profile related to CRC metastasis, we acquired microarray dataset GSE109910 from Gene Expression Omnibus (GEO) and identified 116 differentially expressed lncRNAs (DELs) between metastatic CRC tissue samples and non-metastatic samples with *p*FDR < 0.05 and |LFC|> 1.5 (Fig. 1A, B). *BACE1-AS* was not the highest up-regulated lncRNA in metastatic CRC tissue, however, it ranked most up-regulated lncRNAs associated with poor overall survival among all DELs (Fig. 1C). This observation was confirmed by up-regulated *BACE1-AS* expression in CRC compared to normal tissues from the TCGA database (Fig. 1D). It was further validated in collected liver metastatic and non-metastatic CRC samples (Fig. 1E-G, Table 1), as well as in high metastasis CRC cell lines (SW620, LoVo) and low metastasis CRC cell lines (SW480, HCT116) or CCD841 CoN human normal colonic epithelial cells (Fig. 1H). These results suggest that *BACE1-AS* plays a vital role in CRC liver metastasis.

**Table 1:**
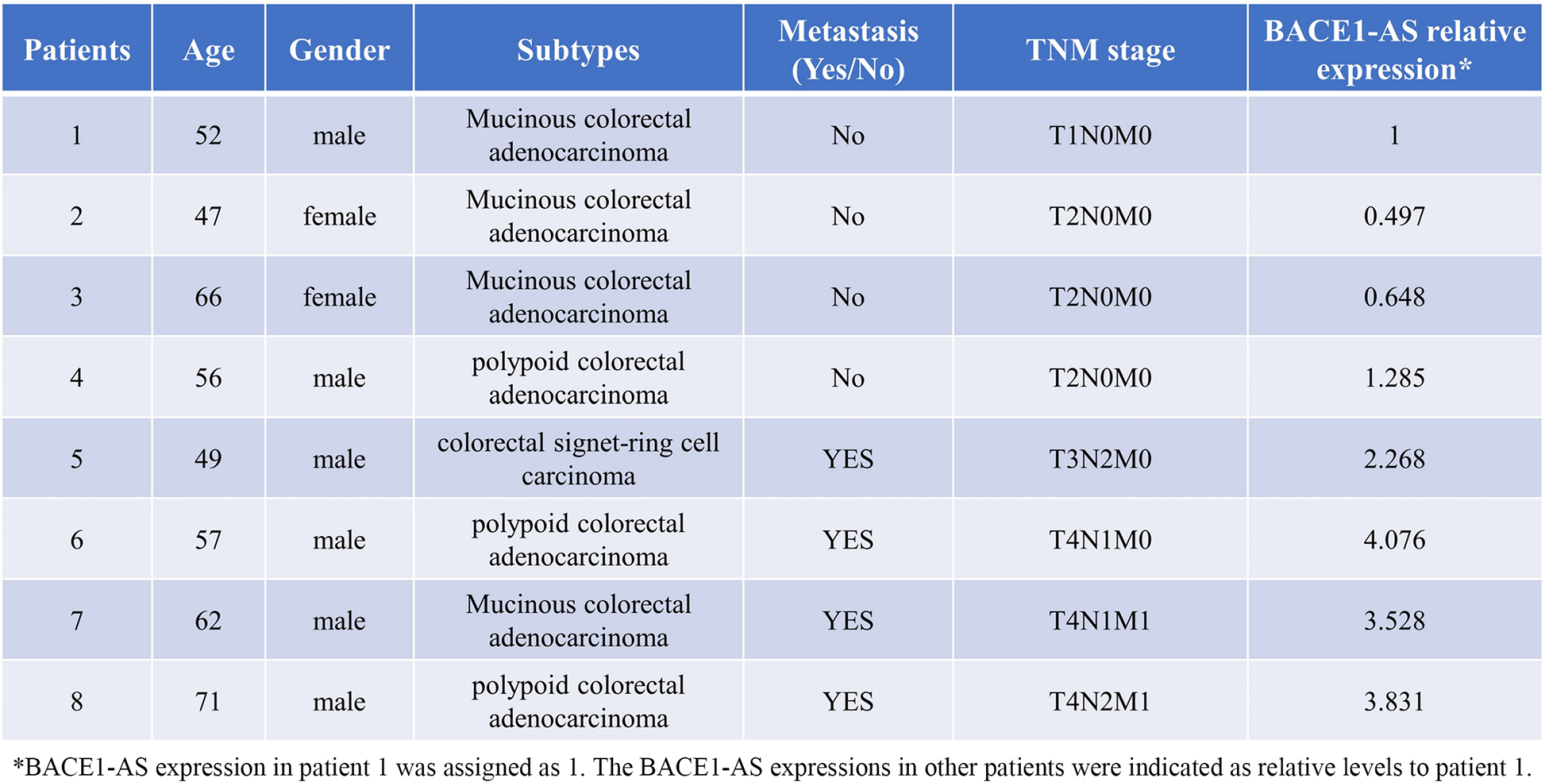
CRC patients’ information

N6-methyladenosine (m6A) modification is considered as conserved internal transcript modification regulating RNA stability [22]. By sequence blast, we found two possible m6A motifs [23] in *BACE1-AS* at 155nt and 790nt (Fig. 1I), suggesting that m6A modification might promote *BACE1-AS* stability and thus increase *BACE1-AS* level in metastatic CRC cells. To test this hypothesis, RNA binding protein immunoprecipitation assay (RIP) with m6A antibody demonstrated approximate 11.2-fold enrichment of *BACE1-AS* over IgG control (Fig. 1J), indicating m6A modification in *BACE1-AS*. Moreover, a significantly higher m6A modification level was found in liver metastatic CRC than in non-metastatic CRC, suggesting that m6A modification was required to boost the *BACE1-AS* level in liver metastatic CRC (Fig. 1K-L). To understand whether these two possible m6A motifs were both modified, we generated single or double site mutant *BACE1-AS* by "A" to "T" mutation (Fig. 1H). Then, SW620 cells transfected with wild-type or mutant *BACE1-AS* expression vector were treated with actinomycin D (2 μg/ml) for different periods before total RNA extraction. As shown in Fig. 1L, *BACE1-AS* decay in single mutant groups was faster than wild-type group, whereas double mutation resulted in an even faster decay than single mutant *BACE1-AS* groups (Fig. 1M), suggesting that two m6A motifs were both required for maintaining *BACE1-AS* stability in CRC cells. These results demonstrated that m6A modifications at 155nt and 790nt caused up-regulation of *BACE1-AS* in CRC liver metastasis.

### IGF2BP2 is required for m6A mediated *BACE1-AS* stabilization

**Fig. 2:**
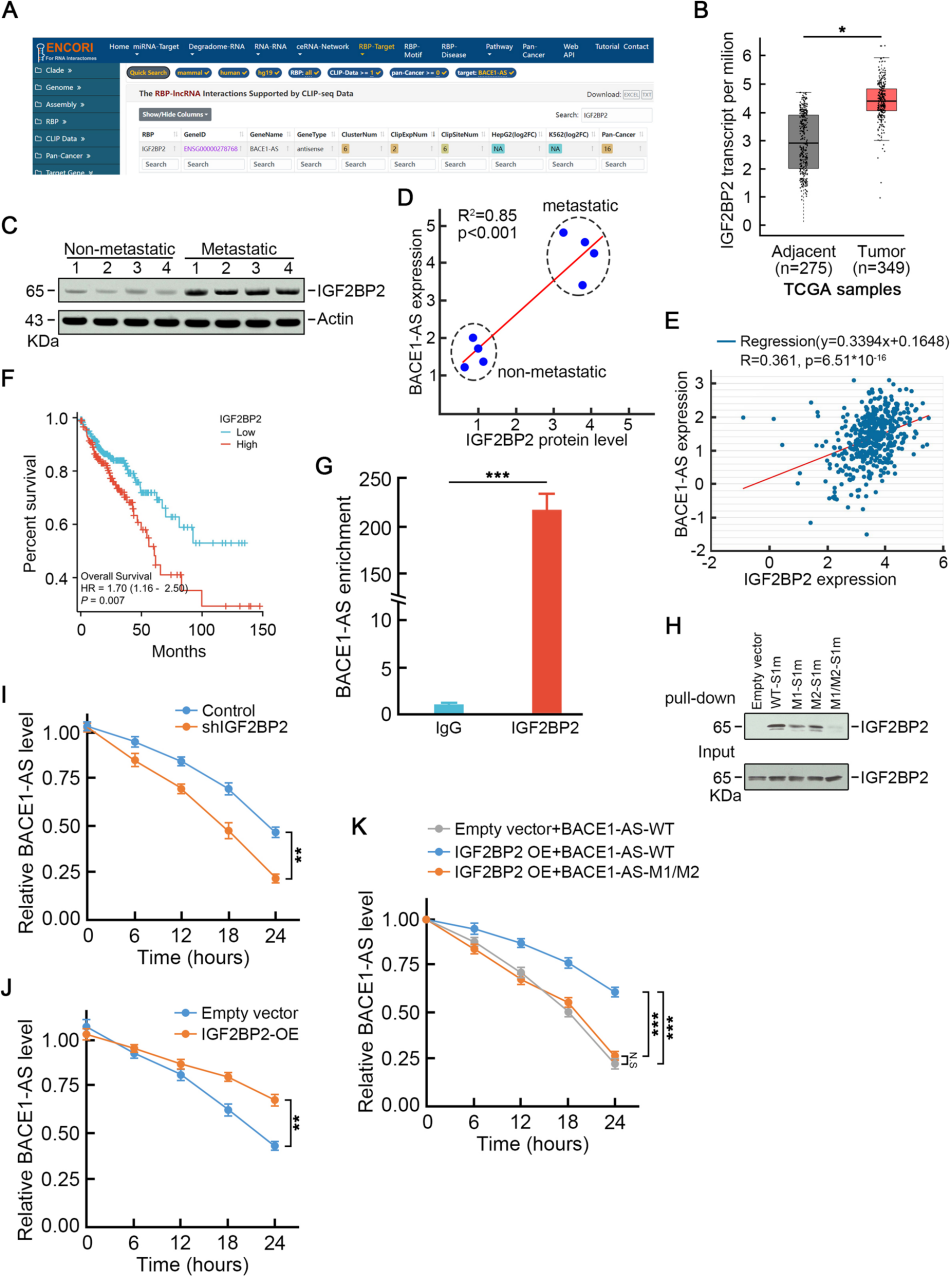
IGF2BP2 binds and promotes *BACE1-AS* stability in metastatic CRC. **A** IGF2BP2 was a potential binding protein of *BACE1-AS* predicted by the ENCORI database. **B** IGF2BP2 was increased in CRC tissues compared to that in adjacent normal tissues. Data from TCGA database. *p < 0.05. **C** IGF2BP2 was up-regulated in collected metastatic CRC samples compared to that in non-metastatic CRC samples. **D** and **E** Positive correlation between BACE1- expression and IGF2BP2 protein level in CRC tissues. Data from collected metastatic and non-metastatic CRC tissues (**D**) and from TCGA database (**E**). **F** Higher IGF2BP2 indicated a poorer prognosis for CRC patients. **G** IGF2BP2 bound to *BACE1-AS* determined by RIP assay using IGF2BP2 antibody. ***p < 0.001. **H** The interaction between IGF2BP2 and *BACE1-AS* was examined using an S1m pull-down assay. S1m tagged wild-type, or mutant *BACE1-AS*, was precipitated using streptavidin beads, and IGF2BP2 was detected by immunoblotting using IGF2BP2 antibody. **I** IGF2BP2 knockdown accelerated *BACE1-AS* decay. **p < 0.01. **J** Over-expression of IGF2BP2 increased *BACE1-AS* stability. **p < 0.01. **K** over-expression of IGF2BP2 increased stability of wild-type *BACE1-AS* rather than mutant *BACE1-AS*. ***p < 0.001

We discovered IGF2BP2, an m6A reader and RNA stabilizer, as a potential *BACE1-AS* binding protein by analyzing the Encyclopedia of RNA Interactomes (ENCORI) database (Fig. 2A). IGF2BP2 was increased in metastatic CRC (Fig. 2B, C) and positively correlated with *BACE1-AS* expression (Fig. 2D, E). Additionally, higher IGF2BP2 level was associated with poorer prognosis (Fig. 2F). RIP assay with IGF2BP2 antibody showed extreme enrichment of *BACE1-AS* compared to IgG control (Fig. 2G). To examine the interaction between *BACE1-AS* and IGF2BP2, we tagged *BACE1-AS* with S1m, a biotin-like aptamer derived from streptavidin-binding aptamer S1 [24, 25]. Then, SW620 cells were transfected with S1m tagged *BACE1-AS*, and the cell extract was subjected to a pull-down assay using streptavidin beads. Immunoblotting with antibodies against IGF2BP2 detected a specific positive band (Fig. 2H), representing a natural IGF2BP2/*BACE1-AS* interaction. However, m6A single mutant in *BACE1-AS* lessened this positive band intensity, and the double mutant caused a negative outcome (Fig. 2H). These observations revealed that IGF2BP2 could bind to *BACE1-AS* through both m6A sites. Next, we examined whether IGF2BP2 protects *BACE1-AS* from RNA decay. Depletion of IGF2BP2 (Fig. S1A) accelerated *BACE1-AS* decay (Fig. 2I), whereas over-expression of IGF2BP2 (Fig. S1B) suppressed *BACE1-AS* decay (Fig. 2J). Moreover, m6A motif mutation abolished IGF2BP2 protective effect on *BACE1-AS* (Fig. 2K). These data further supported that IGF2BP2 was required for m6A-mediated *BACE1-AS* stabilization.

### *BACE1-AS* promotes CRC liver metastasis

**Fig. 3:**
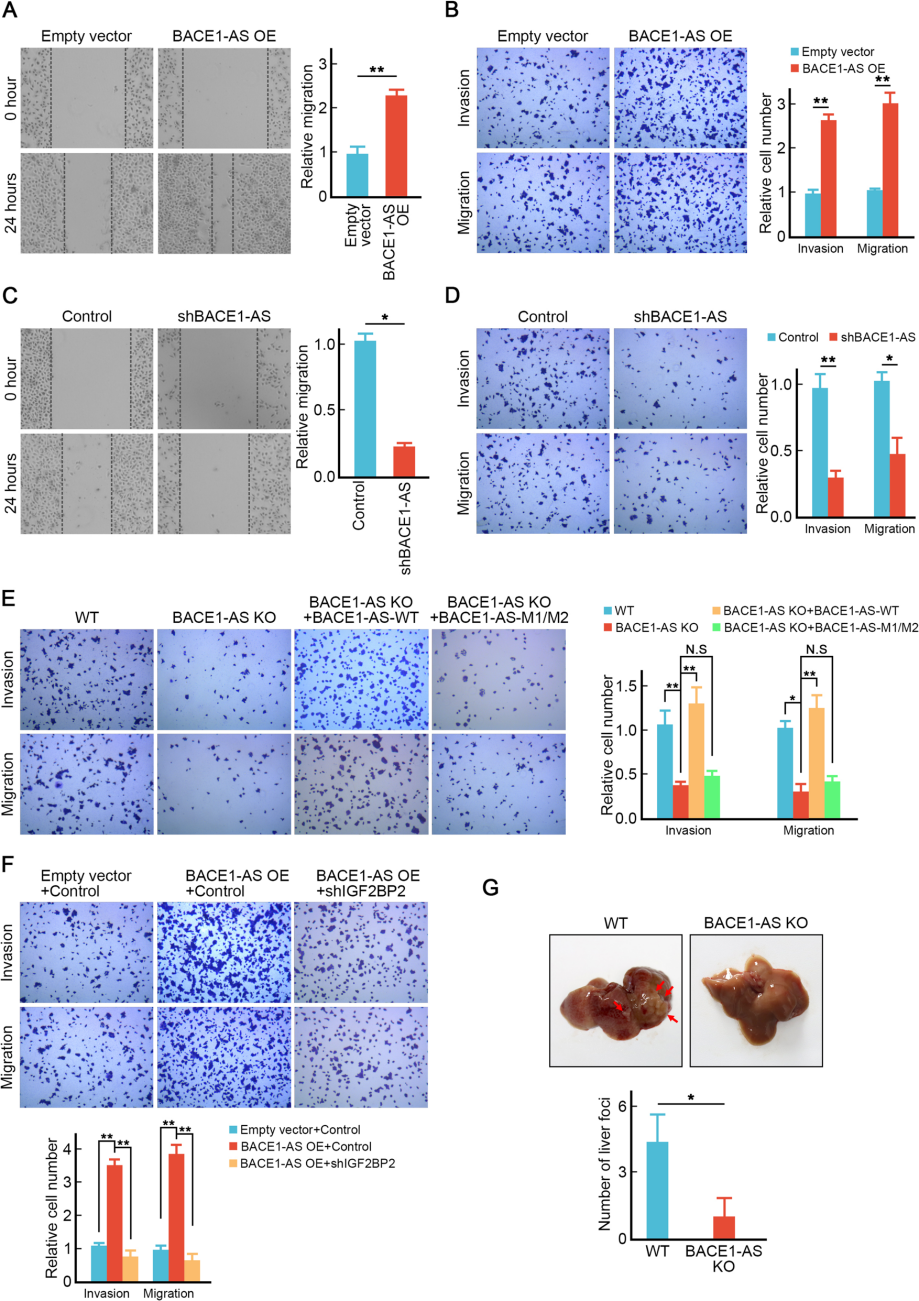
*BACE1-AS* promotes CRC metastasis in vitro and in vivo. **A** *BACE1-AS* over-expression promoted wound healing speed in HCT116 cells. Three individual experiments' representative images show similar results (left panel). The bar graph shows the mean healing speed (right panel). *p < 0.05. **B** *BACE1-AS* promoted migration and invasion in HCT116 cells. Representative trans-well assay images are from three individual experiments showing similar results (left panel). The bar graph shows relative numbers of migration and invasion cells counted from three individual experiments (right panel). *p < 0.05. **C** Depletion of *BACE1-AS* inhibited wound healing ability in SW620 cells. Representative images are from three individual experiments showing similar results (left panel). The bar graph shows the mean healing speed (right panel). *p < 0.05. **D** Knock-down of *BACE1-AS* inhibited migration and invasion in SW620 cells. Representative trans-well assay images are from three individual experiments showing similar results (left panel). The bar graph shows relative numbers of migration and invasion cells counted from three individual experiments (right panel). *p < 0.05. **E** *BACE1-AS* knockout led to reduction in cell invasion and migration which could be rescued by exogenous expression of wild-type *BACE1-AS* but not by m6A motifs mutant *BACE1-AS*. Representative images are from three individual experiments showing similar results (left panel). The bar graph shows relative numbers of migration and invasion cells counted from three individual experiments (right panel). *p < 0.05. **F** Depletion of IGF2BP2 suppressed capabilities of migration and invasion in *BACE1-AS* over-expression cells. Representative images are from three individual experiments showing similar results (left panel). The bar graph shows relative numbers of migration and invasion cells counted from three individual experiments (right panel). *p < 0.05. **G** *BACE1-AS* knockout suppressed CRC liver metastasis in vivo

In order to examine the biological roles of *BACE1-AS* in CRC liver metastasis, HCT116 cells infected with lentivirus carrying *BACE1-AS* or empty virus (Fig. S2A) were subjected to metastasis assays. The wound healing assay demonstrated a faster healing speed upon *BACE1-AS* over-expression (Fig. 3A). In addition, the Transwell assay revealed higher migration and invasion capabilities in *BACE1-AS* over-expressed cells (Fig. 3B). In contrast, the knockdown of *BACE1-AS* by lentivirus infection carrying shRNA targeting *BACE1-AS* (Fig. S2B) suppressed cell migration and invasion in SW620 cells (Fig. 3C, D).

We generated *BACE1-AS* knockout SW620 cells to better characterize the effects of *BACE1-AS* in CRC metastasis (Fig. S3). *BACE1-AS* knockout led to a significant reduction in cell invasion and migration (Fig. 3E). Notably, these declined metastasis phenotypes could be rescued by exogenous expression of wild-type *BACE1-AS* (Fig. 3E). However, m6A motifs mutant *BACE1-AS* could not restore the metastasis abilities in *BACE1-AS* knockout cells (Fig. 3E), suggesting that m6A modification was essential for *BACE1-AS* promoted CRC liver metastasis. Interestingly, depletion of IGF2BP2 abolished *BACE1-AS* over-expressed promoted metastasis enhancement in *BACE1-AS* knockout cells (Fig. 3F).

Next, *BACE1-AS* knockout SW620 cells and parental SW620 cells were injected into the spleens of NOD SCID mice to investigate the role of *BACE1-AS* in promoting CRC liver metastasis in vivo. After 6-weeks of housing, the mice were sacrificed, and the number of metastatic nodules in the liver was counted. As expected, NOD SCID mice injected with parental SW620 generated a certain number of metastatic nodules in the liver (Fig. 3G). However, loss of *BACE1-AS* significantly suppressed SW620 cell metastasis characterized by a smaller number of metastatic nodules in the liver (Fig. 3G). These data indicated that *BACE1-AS* played a critical role in CRC liver metastasis.

### *BACE1-AS* promotes stemness-like properties in CRC cells

**Fig. 4:**
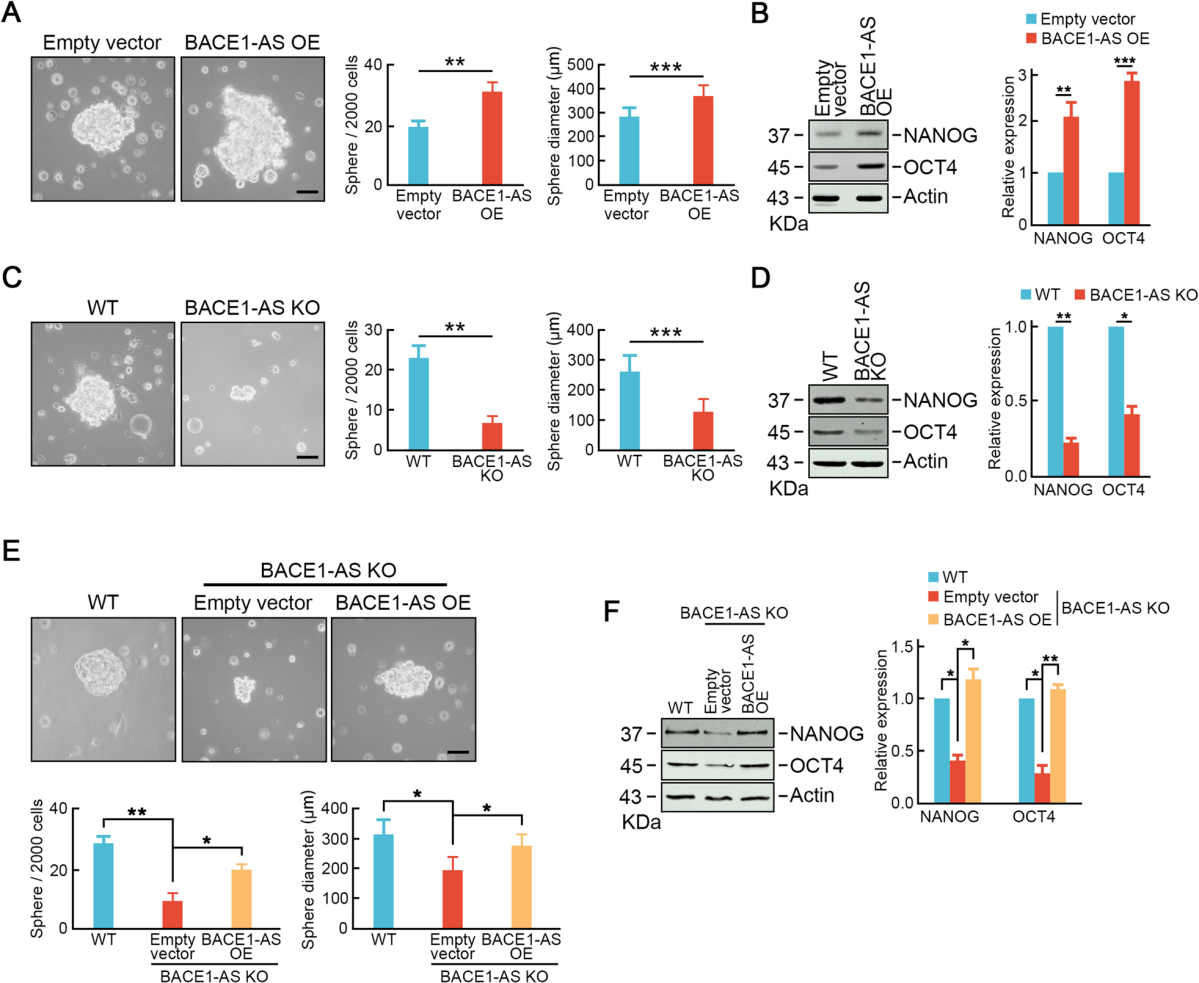
*BACE1-AS* promotes stemness-like properties in CRC cells. **A** Over-expression of *BACE1-AS* increased tumor sphere formation ability in SW620 cells. Representative images are shown (left panel). Bar graphs show the mean numbers (middle panel) and diameters (right panel) of tumorspheres. Scale bar = 100 μm. *p < 0.05, ***p < 0.001. **B** Enforced expression of *BACE1-AS* promoted the expressions of stemness-related factors, including NANOG and OCT4. Actin served as a loading control. Representative blots from three experiments are shown (left panel). Ratios of levels of NANOG and OCT4 vs. Actin were calculated using NIH Image J 1.61 (right panel). **p < 0.01. **C** *BACE1-AS* knockout SW620 cells exhibited lower tumor sphere formation ability than parental cells. Representative images are shown (left panel). Bar graphs show the mean numbers (middle panel) and diameters (right panel) of tumorspheres. Scale bar = 100 μm. *p < 0.05, ***p < 0.001. **D** *BACE1-AS* knockout suppressed the expressions of NANOG and OCT4. Actin served as a loading control. Representative blots from three experiments are shown (left panel). Ratios of levels of NANOG and OCT4 vs. Actin were calculated using NIH Image J 1.61 (right panel). **p < 0.01. **E** Ectopic expression of *BACE1-AS* rescued tumorsphere formation ability in *BACE1-AS* knockout cells. Representative images are shown (left panel). Bar graphs show the mean numbers (middle panel) and diameters (right panel) of tumorspheres. Scale bar = 100 μm. *p < 0.05, ***p < 0.001. **F** Ectopic expression of *BACE1-AS* restored expressions of NANOG and OCT4. Actin served as a loading control. Representative blots from three experiments were shown (left panel). Ratios of levels of NANOG and OCT4 vs. Actin were calculated using NIH Image J 1.61 (right panel). **p < 0.01

Cancer stem cells have been proposed to fuel CRC metastasis. Thus, we further asked whether *BACE1-AS* promoted stemness-like properties in CRC cells. Ectopic expression of *BACE1-AS* by lentivirus infection remarkably increased the tumor sphere formation in sphere numbers and sphere diameters compared to control cells (Fig. 4A). Consistently, *BACE1-AS* over-expression significantly increased the protein levels of stemness-related factors, including NANOG and OCT4 (Fig. 4B). In contrast, *BACE1-AS* knockout cells exhibited weaker capabilities of tumor sphere formation (Fig. 4C) and reduced protein levels of stemness-related factors (Fig. 4D). In addition, restoration of *BACE1-AS* in *BACE1-AS* knockout cells rescued tumor sphere formation capabilities and expression of NANOG, OCT4 (Fig. 4E, F). These data suggested that *BACE1-AS* promoted stemness-like properties in CRC cells, which could contribute to CRC liver metastasis.

### *BACE1-AS* promotes CRC liver metastasis through TUFT1

**Fig. 5:**
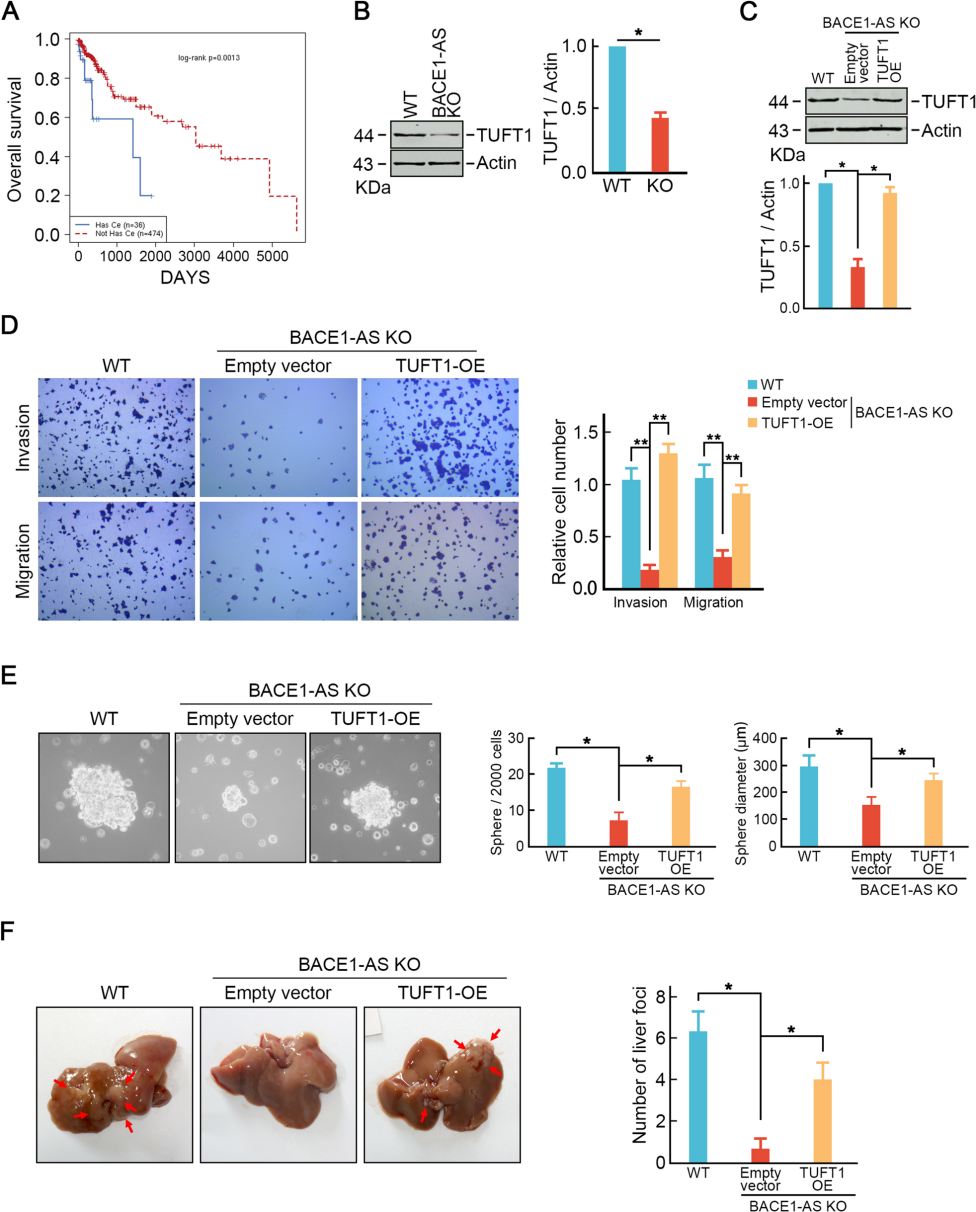
*BACE1-AS* promotes CRC metastasis through TUFT1. **A** CRC patients with *BACE1-AS*/TUFT1 ceRNA network suffered shorter overall survival. **B** TUFT1 expression was decreased in *BACE1-AS* knockout cells. Actin served as a loading control. Representative blots from three experiments were shown (left panel). Ratios of the level of TUFT1 vs. Actin were calculated using NIH Image J 1.61 (right panel). **p < 0.01. **C** Ectopic expression of TUFT1 restored TUFT1 level in *BACE1-AS* knock-out cells. Actin served as a loading control. Representative blots from three experiments are shown (upper panel). Ratios of the level of TUFT1 vs. Actin were calculated using NIH Image J 1.61 (lower panel). **p < 0.01. **D** Ectopic expression of TUFT1 restored metastasis abilities in *BACE1-AS* knockout cells determined by trans-well assay. **E** Exogenous expression of TUFT1 rescued tumor sphere formation ability in *BACE1-AS* knockout cells. Representative images are shown (left panel). Bar graphs show the mean numbers (middle panel) and diameters (right panel) of tumorspheres. **F** Exogenous expression of TUFT1 restored liver metastasis capability in *BACE1-AS* knockout cell

Acting as competing endogenous RNAs (ceRNA) to promote target mRNA expression is a key mechanism of lncRNAs. LncACTdb 3.0 database identified a potential BACE1/TUFT1 ceRNA network axis [26]. CRC patients with *BACE1-AS*/TUFT1 axis exhibited shorter overall survival than those without this ceRNA network (Fig. 5A). Furthermore, TUFT1 was found to be up-regulated in CRC cells, while high metastasis potential CRC cells harbored even higher TUFT1 protein level (Fig. S4A), which was positively correlated with *BACE1-AS* expression (Fig. S4B). This observation was also found in metastatic CRC tissues than non-metastatic tissues and normal tissues (Fig. S4C-F). Additionally, higher TUFT1 indicated unfavorable prognostic outcomes (Fig. S4G). Depletion of TUFT1 inhibited migration and invasion capabilities in SW620 cells (Fig. S4H). Moreover, TUFT1 loss suppressed tumor sphere formation and stemness-related factors expression (Fig. S4I-J). These phenotypes caused by TUFT1 depletion were consistent with *BACE1-AS* loss, raising the possibility that *BACE1-AS* promoted CRC cell liver metastasis through promoting TUFT1.

To test this hypothesis, we examined TUFT1 expression in *BACE1-AS* knockout cells. As expected, TUFT1 expression was remarkably decreased in *BACE1-AS* knockout cells compared to parental cells (Fig. 5B). Exogenous expression of TUFT1 (Fig. 5C) restored migration and invasion capabilities in *BACE1-AS* knockout SW620 cells (Fig. 5D). Furthermore, TUFT1 over-expression also rescued stemness-like properties in *BACE1-AS* knockout SW620 cells analyzed by the number and size of formed tumor spheres (Fig. 5E). Importantly, TUFT1 over-expression also restored *BACE1-AS* knockout cells liver metastasis in vivo (Fig. 5F). These data further indicated that *BACE1-AS* promoted CRC cell liver metastasis through TUFT1.

### *BACE1-AS* promotes TUFT1 dependent Wnt signaling activation

**Fig. 6:**
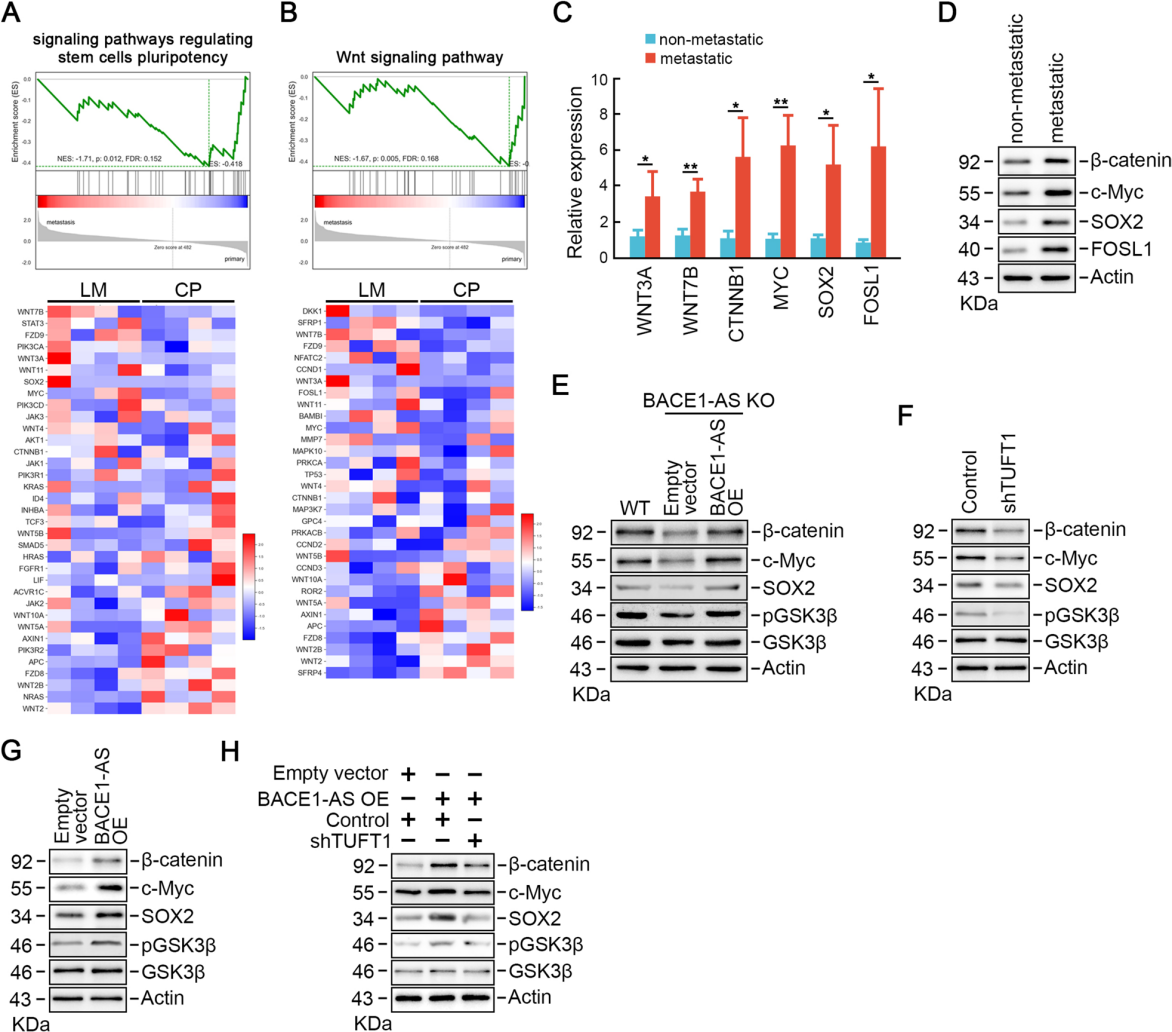
*BACE1-AS* promotes TUFT1 dependent Wnt signaling activation. **A** and **B** GSEA plots (upper panel) and heatmap (lower panel) depicting microarray analysis of mRNAs encoding proteins involved in signaling pathways regulating pluripotency of stem cells (**A**) and Wnt signaling pathway (**B**) in liver metastatic or primary CRC tissues. LM: liver metastasis; CP: CRC primary. **C** Confirmation of the top microarray targets WNT3A, WNT7B, CTNNB1, MYC, SOX2 and FOSL1 by RT-qPCR in collected liver metastatic and non-metastatic CRC tissues. *p < 0.05, **p < 0.01. **D** Protein levels of β-catenin, Myc, SOX2 and FOSL1 in collected liver metastatic and non-metastatic CRC tissues. **E** Knockout of *BACE1-AS* inactivated Wnt signaling pathway could be rescued by exogenous expression of *BACE1-AS*. **F** TUFT1 depletion decreased GSK3β phosphorylation and the protein levels of β-catenin, SOX2 and c-Myc. **G** TUFT1 over-expression increased GSK3β phosphorylation and the protein levels of β-catenin, SOX2 and c-Myc. **H** TUFT1 depletion reversed *BACE1-AS* over-expression induced Wnt signaling pathway hyper-activation

To explore the *BACE1-AS* regulated signaling pathways involved in CRC liver metastasis, we acquired microarray dataset GSE224235 compering the matched primary and liver metastatic lesions, and identified several signaling pathways regulating pluripotency of stem cells were critical for CRC liver metastasis, among which was Wnt signaling pathway (Fig. 6A-B). qRT-PCR analysis confirmed that the up-regulation profiles of mRNAs encoding proteins involved in Wnt signaling pathway including WNT3A, WNT7B, CTNNB1, MYC, SOX2 and FOSL1, in metastatic CRC cells compared to non-metastatic CRC samples (Fig. 6C). Western blot analysis demonstrated that the protein levels of β-catenin, c-Myc, SOX2 and FOSL1 reflected the consistent alterations in mRNA levels (Fig. 6D).

It is well documented that Wnt signaling pathway is critical for cancer metastasis and stemness-like property maintenance [27–29]. Thus, we asked whether the pro-metastatic functions of *BACE1-AS* were dependent on Wnt signaling pathways. *BACE1-AS* knockout remarkably decreased the phosphorylation of GSK3β as well as the protein levels of β-catenin and Wnt signaling downstream targets SOX2 and c-Myc (Fig. 6E). Moreover, *BACE1-AS* restoration rescued the reduced levels of β-catenin, SOX2 and c-Myc in *BACE1-AS* knockout cells (Fig. 6E). Interestingly, TUFT1 knockdown suppressed the phosphorylation of GSK3β, and subsequently decreased the protein levels of Wnt signaling downstream targets (Fig. 6F), while TUFT1 over-expression exhibited opposite effects (Fig. 6G), indicating that TUFT1 is an upstream regulator of Wnt signaling pathway. Notably, *BACE1-AS* over-expression induced Wnt signaling pathway hyper-activation could be reversed by TUFT1 depletion (Fig. 6H), indicating that *BACE1-AS* activated Wnt signaling pathway in a TUFT1 dependent manner.

### Pharmacologic inhibition of Wnt signaling suppresses *BACE1-AS* promoted CRC stemness-like properties and liver metastasis

**Fig. 7:**
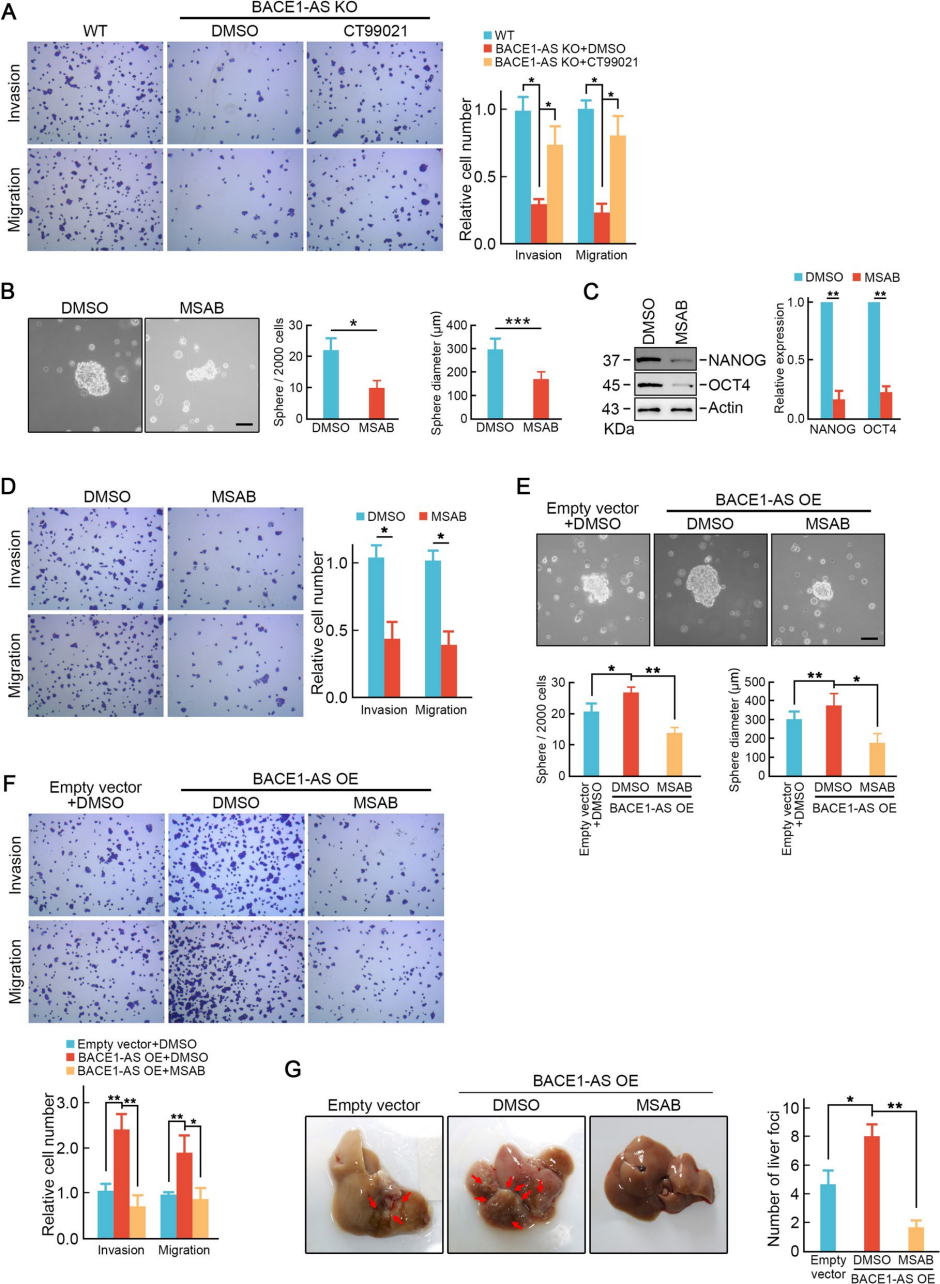
Pharmacologic inhibition of Wnt signaling suppresses *BACE1-AS* promoted CRC stemness-like properties and liver metastasis. **A** Reactivation of Wnt signaling by CT99021 treatment reversed capabilities of migration and invasion in *BACE1-AS* knockout cells. **B** MSAB treatment inhibited tumor sphere formation in SW620 cells. *p < 0.05. **C** MSAB treatment decreased the protein levels of NANOG and OCT4. **p < 0.01. **D** MSAB treatment suppressed capabilities of migration and invasion in SW620 cells. *p < 0.05. **E** MSAB treatment inhibited tumor sphere formation in *BACE1-AS* over-expressed cells. *p < 0.05, **p < 0.01. **F** MSAB treatment suppressed capabilities of migration and invasion in *BACE1-AS* over-expressed cells. *p < 0.05, **p < 0.01. **G** Inactivation of Wnt signaling repressed *BACE1-AS* over-expression promoted CRC liver metastasis in vivo. *p < 0.05, **p < 0.01

Next, we investigated whether activation of Wnt signaling pathway is essential for *BACE1-AS* promoting CRC liver metastasis. As expected, re-activation of Wnt signaling pathway by CT99021 treatment (10 μM) rescued metastatic potential loss in *BACE1-AS* knockout SW620 cells (Fig. 7A). Pharmacologic inhibition of Wnt signaling pathway by MSAB (10 μM) significantly suppressed tumor sphere formation in SW620 cells and the expression of stemness-related factors NANOG and OCT4 (Fig. 7B-C). MSAB treatment also inhibited the capabilities of migration and invasion in SW620 cells (Fig. 7D). More importantly, MSAB treatment blocked tumor sphere formation and capabilities of migration and invasion in *BACE1-AS* over-expressed cells (Fig. 7E-F). In vivo experiments demonstrated that MSAB treatment (20 mg/kg) repressed *BACE1-AS* over-expression promoted CRC liver metastasis in vivo (Fig. 7G).

Taken together, these results suggested that activation of Wnt signaling is vital for *BACE1-AS* promoting liver metastasis. And pharmacologic inhibition of Wnt signaling suppresses *BACE1-AS* promoted CRC stemness-like properties and liver metastasis both in vitro and in vivo.

### *BACE1-AS* enhances TUFT1 expression through sponging miR-214-3p

**Fig. 8:**
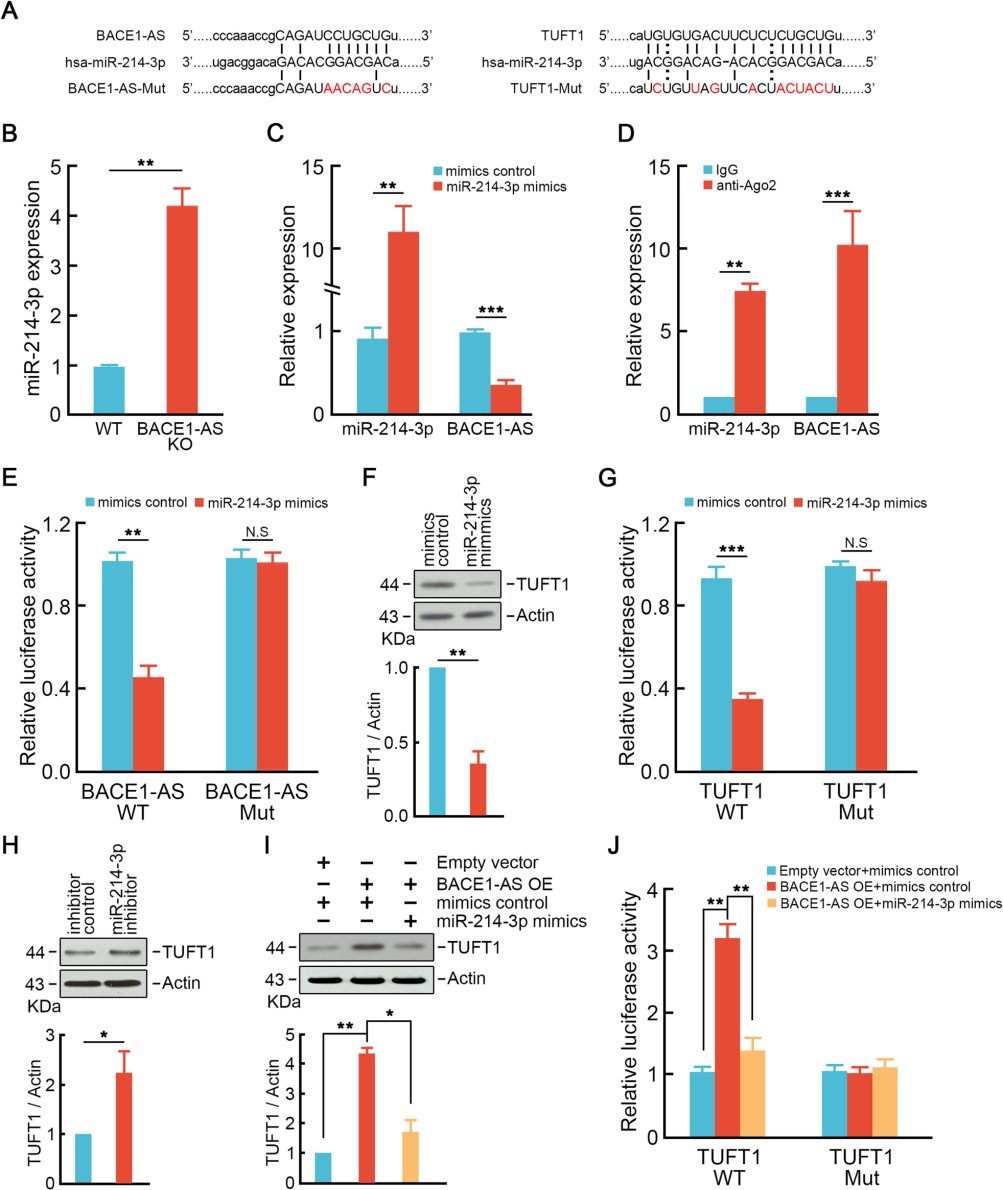
*BACE1-AS* enhances TUFT1 expression through sponging miR-214-3p. **A** Schematic diagram of miR-214-3p potential binding sites on *BACE1-AS* and TUFT1. **B** *BACE1-AS* knockout significantly increased miR-214-3p expression. **p < 0.01. **C** Over-expression of miR-214-3p suppressed *BACE1-AS* expression. **p < 0.01, ***p < 0.001. **D** Relative enrichments of *BACE1-AS* and miR-214-3p in Ago2 immunoprecipitated pellets were examined by RIP assay using anti-Ago2 antibody followed by qRT-PCR. **p < 0.01, ***p < 0.001. **E** MiR-214-3p mimics inhibited luciferase activity in wild-type *BACE1-AS* groups rather than in mutant *BACE1-AS* groups. **p < 0.01. **F** MiR-214-3p suppressed TUFT1 expression. Representative blots were shown (upper panel). Actin served as a loading control. Ratios of levels of TUFT1 vs. Actin was calculated using NIH Image J 1.61 (right panel). **p < 0.01. **G** MiR-214-3p mimics inhibited luciferase activity in wild-type TUFT1 groups rather than in mutant TUFT1 groups. ***p < 0.001. **H** MiR-214-3p inhibitor suppressed TUFT1 expression in *BACE1-AS* knockout cells. Representative blots are shown (upper panel). Actin served as a loading control. Ratios of levels of TUFT1 vs. Actin was calculated using NIH Image J 1.61 (right panel). *p < 0.05. **I** over-expression of miR-214-3p reversed *BACE1-AS* induced TUFT1 up-regulation. Representative blots were shown (upper panel). Actin served as a loading control. Ratios of levels of TUFT1 vs. Actin was calculated using NIH Image J 1.61 (right panel). *p < 0.05, **p < 0.01. **J** MiR-214-3p mimics reversed *BACE1-AS* over-expression-induced luciferase activity increasing in wild-type TUFT1 groups rather than in TUFT1 mutant groups. **p < 0.01

Since we found *BACE1-AS* promoted CRC cells liver metastasis through TUFT1 and a potential ceRNA network was predicted, we next tried to establish this ceRNA network between *BACE1-AS* and TUFT1. ENCORI database predicted a miR-214-3p binding site both in *BACE1-AS* and TUFT1 (Fig. 8A). MiR-214-3p was significantly decreased in CRC compared to normal tissues (Fig. S5A). Additionally, the miR-214-3p level in high metastasis potential CRC cells was even lower than that in low metastasis potential CRC cells (Fig. S5B, C). *BACE1-AS* knockout dramatically increased miR-214-3p expression (Fig. 8B). In contrast, miR-214-3p mimics suppressed *BACE1-AS* expression (Fig. 8C). RIP assay showed remarkable enrichments of *BACE1-AS* and miR-214-3p in precipitated pellets from SW620 cells by Ago2 antibody immunoprecipitation compared to IgG pellets (Fig. 8D). Further, dual luciferase reporter assay was employed to test the direct interaction between *BACE1-AS* and miR-214-3p. In line with the RIP assay, we found that miR-214-3p mimics significantly repressed the luciferase activity in the *BACE1-AS*-WT group but did not alter the luciferase activity in binding sites mutant groups (Fig. 8E).

Next, we examined whether *BACE1-AS* regulated TUFT1 expression through miR-214-3p. Over-expression of miR-214-3p inhibited TUFT1 expression (Fig. 8F). Luciferase reporter assay demonstrated that miR-214-3p mimics significantly reduced the luciferase activity of TUFT1-WT but did not alter the luciferase activity in binding sites mutant groups (Fig. 8G). In *BACE1-AS* knockout cells, the miR-214-3p inhibitor could rescue TUFT1 expression (Fig. 8H). Besides, over-expression of *BACE1-AS* induced miR-214-3p could reverse TUFT1 up-regulation mimics co-transfection (Fig. 8I). Likewise, the luciferase reporter assay exhibited similar alterations. MiR-214-3p mimics eliminated the promoting effect of *BACE1-AS* over-expression on luciferase activity in the TUFT1-WT group (Fig. 8J). These findings demonstrated that *BACE1-AS* promotes TUFT1 expression through sponging miR-214-3p.

## Discussion

Metastasis causes the major death of CRC, highlighting an essential requirement in investigating novel markers and targets for effective anti-metastasis therapeutic strategies development [30]. *BACE1-AS* was previously reported to be implicated in neurodegenerative diseases, such as Alzheimer's disease [20, 21] and Parkinson's disease [31]. Recently, some studies have reported that *BACE1-AS* also plays an oncogenic role in hepatocellular carcinoma and breast cancers [13, 32, 33]. In our study using a microarray dataset from CRC tissues with and without metastasis, we identified a group of aberrantly expressed lncRNA, among which *BACE1-AS* is one of the most highly expressed lncRNA promoting CRC liver metastasis and poor prognosis through regulating TUFT1/Wnt signaling axis.

Interestingly, one study that we cannot ignore reported down-regulated *BACE1-AS* in 5-fluorouracil resistance colon cancer cells [34]. In contrast, our results from various sources including TCGA database, different high metastatic CRC cell lines, and cancer tissues from CRC liver metastasis patients, illustrated a significantly higher *BACE1-AS* than non- or low-metastatic CRC. Such contradiction expression pattern of *BACE1-AS* could be explained by different pathological states and models used [35] and also reflects the complexity of *BACE1-AS* regulation in CRC. Another example of the complicated biological roles of lncRNAs could be *MALAT1*. *MALAT1* is previously known as a metastasis-promoting lncRNA but is extremely low in highly aggressive/metastatic breast cancers exhibiting anti-metastasis function [36].

M6A modification is the most widespread post-transcriptional reversible internal modification of eukaryotic lncRNAs [37, 38]. Modified m6A site can be recognized by multiple m6A readers and subsequently regulates the stability or decay of lncRNAs [39, 40]. *MALAT1* was increased in non-small cell lung cancer due to METTL3-mediated high m6A modification. Higher levels of *MALAT1* induce drug resistance and metastasis [41]. LncRNA *FAM225A* is highly m6A enriched, leading to its stabilization in nasopharyngeal carcinoma [42]. On the contrary, YTHDF3 facilitates m6A-modified lncRNA *GAS5* degradation [43]. Thus, the functional roles of m6A are lncRNA specific or might be cancer-specific. We found that the enrichment of m6A in *BACE1-AS* in liver metastatic CRC was remarkably higher than in normal tissues or non-metastatic CRC, revealing a critical post-regulatory role of m6A in elevating the *BACE1-AS* level. Further, m6A-modified *BACE1-AS* was recognized by IGF2BP2, a well-known m6A reader [44], causing an even higher level of *BACE1-AS* in metastatic CRC. These interrelated mechanisms secured an enhanced level of *BACE1-AS*, facilitating CRC liver metastasis.

Cancer stem cells (CSCs) are subpopulations of cancer cells with increased renewal capacity and the ability to recapitulate heterogeneity [45]. Numerous studies have uncovered the unique biological function of CSCs supporting the remodeling of metastatic microenvironments and colonization in distant organs [46–48]. We showed that *BACE1-AS* promoted stemness-like properties in CRC cells. Loss of suppressed tumorsphere ability and the expressions of stemness-related factors. Correspondingly, GSEA assay revealed many signaling pathways regulating pluripotency of stem cells, particular Wnt signaling pathway was changed in liver metastatic CRC compared to primary CRC. *BACE1-AS* promoted CRC liver metastasis through activation of Wnt signaling pathway in a TUFT1 dependent manner. These exciting observations further supported our hypothesis of *BACE1-AS* promoting CRC liver metastasis. Notably, ectopic expression of TUFT1 rescued stemness-like properties and metastatic abilities in *BACE1-AS* knockout cells, indicating that TUFT1 was essential for *BACE1-AS*-induced CRC liver metastasis.

## Conclusion

Our study unveiled a fundamental role for *BACE1-AS* in CRC liver metastasis. IGF2BP2 binding to m6A-modified sites was required to keep *BACE1-AS* levels high in metastatic CRC. Furthermore, increased *BACE1-AS* further promoted CRC liver metastasis through TUFT1/Wnt signaling axis. These findings provided a novel insight into CRC liver metastasis and suggested that *BACE1-AS* and its downstream Wnt signaling pathways could be a promising therapeutic target for metastatic CRC.

### Supplementary Information

**Additional file 1:** **Figure S1.** The efficiencies of IGF2BP2 over-expression and knockdown in CRC cell lines. (A) IGF2BP2 over-expression vector successfully up-regulated IGF2BP2 protein level in HCT116 cells. (B) Transfection of IGF2BP2 shRNA inhibited IGF2BP2 expression in SW620 cells.

**Additional file 2:** **Figure S2.** The efficiencies of *BACE1-AS* over-expression and knockdown in CRC cell lines. (A) *BACE1-AS* over-expression vector successfully up-regulated *BACE1-AS* level in HCT116 cells. (B) Transfection of *BACE1-AS* shRNA inhibited *BACE1-AS* expression in SW620 cells.

**Additional file 3:** **Figure S3.** Schematic diagram of *BACE1-AS* knockout strategy.

**Additional file 4:** **Figure S4.** TUFT1 promotes CRC metastasis. (A) CRC cell lines expressed higher TUFT1 than CCD841 CoN normal human colonic epithelial cell line. (B) Positive correlation between *BACE1-AS* and TUFT1. Data from TCGA database. (C) Elevated TUFT1 was found in CRC tissues. Data from TCGA database. ***p<0.001. (D) TUFT1 levels in collected non-metastatic and metastatic CRC were determined by immunoblotting. (E-F) TUFT1 expression was increased in M stage (E) and N stage (F). M1 stage and N1/N2 stage tissues harbored even higher TUFT1 expression. ***p<0.001. (G) Higher TUFT1 level indicated worse CRC overall survival. (H) Knockdown of TUFT1 suppressed abilities of invasion and migration in SW620 cells. Representative images are shown (left panel). The bar graph shows the relative numbers of migration and invasion cells counted from three individual experiments (right panel). ***p<0.001. (I) Depletion of TUFT1 inhibited tumor sphere formation in SW620 cells. Scale bar = 100μm. *p<0.05, ***p<0.001. (J) Knockdown of TUFT1 suppressed the expressions of NANOG and OCT4 in SW620 cells. Actin served as a loading control. Representative blots from three experiments are shown (left panel). Ratios of levels of NANOG and OCT4 vs. Actin were calculated using NIH Image J 1.61 (right panel). **p<0.01.

**Additional file 5:** **Figure S5.** miR-214-3p is increased in metastatic CRC. (A) miR-214-3p was significantly decreased in CRC samples compared to normal tissues. Data from TCGA database. ***p<0.001. (B) miR-214-3p was significantly decreased in CRC cell lines compared to CCD841 CoN normal human colonic epithelial cell line. High metastasis potential cells (SW620 and LoVo) harbored even lower miR-214-3p than low metastasis potential cells (SW480 and HCT116). **p<0.01, ***p<0.001. (C) miR-214-3p expression in collected metastatic and non-metastatic CRC samples was examined by qRT-PCR. **p<0.01.

## Data Availability

The datasets generated during and/or analyzed in this study are available from the corresponding authors Xidi Wang and Zizhen Si.

## Abbreviations

CRC: Colorectal cancer
LncRNA: Long non-coding RNA
*BACE1-AS*: Beta-secretase 1 antisense RNA
RIP: RNA Binding protein immunoprecipitation
TCGA: The cancer genome atlas
IGF2BP2: Insulin like growth factor 2 mRNA binding protein 2
NANOG: Nanog homeobox
OCT4: POU Class 5 homeobox 1
ceRNA: Competing endogenous RNA
TUFT1: Tuftelin 1
ANOVA: Analysis of variance
