# Supplementary figures and images for: m6A modified BACE1-AS contributes to liver metastasis and stemness-like properties in colorectal cancer through TUFT1 dependent activation of Wnt signaling
Source: J Exp Clin Cancer Res. 2023 Nov 21;42:306. doi: 10.1186/s13046-023-02881-0 (PMC10661562; doi:10.1186/s13046-023-02881-0)

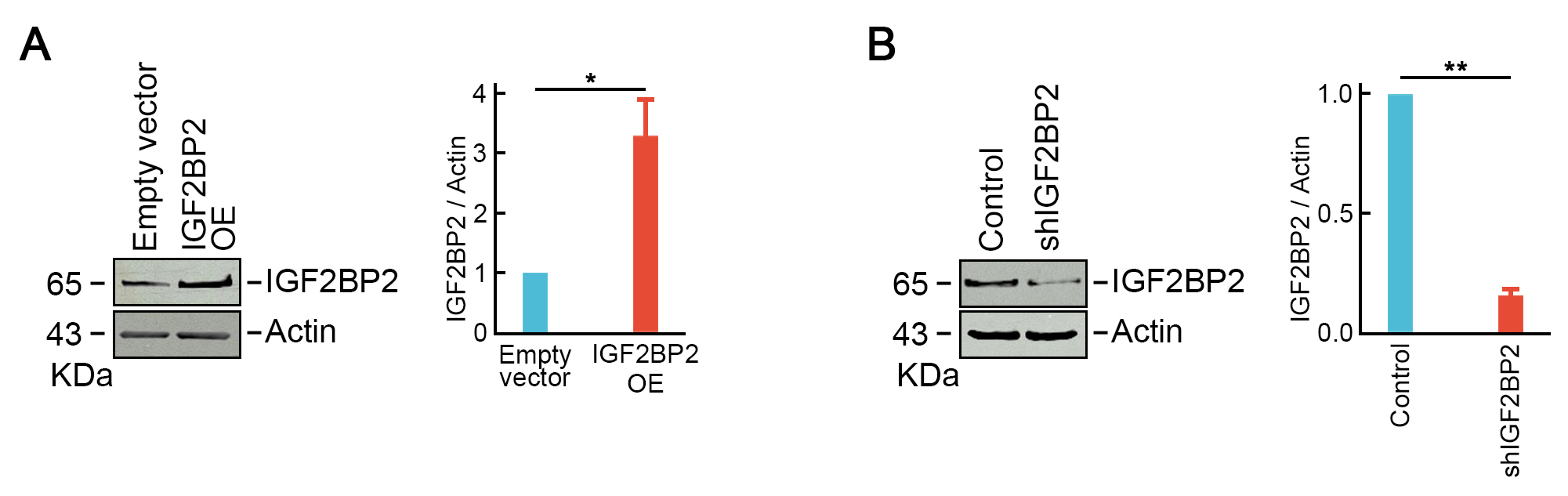

Supplement: Supplementary file 1 — Additional file 1: Figure S1. The efficiencies of IGF2BP2 over-expression and knockdown in CRC cell lines. (A) IGF2BP2 over-expression vector successfully up-regulated IGF2BP2 protein level in HCT116 cells. (B) Transfection of IGF2BP2 shRNA inhibited IGF2BP2 expression in SW620 cells. [file 13046_2023_2881_MOESM1_ESM.jpg]

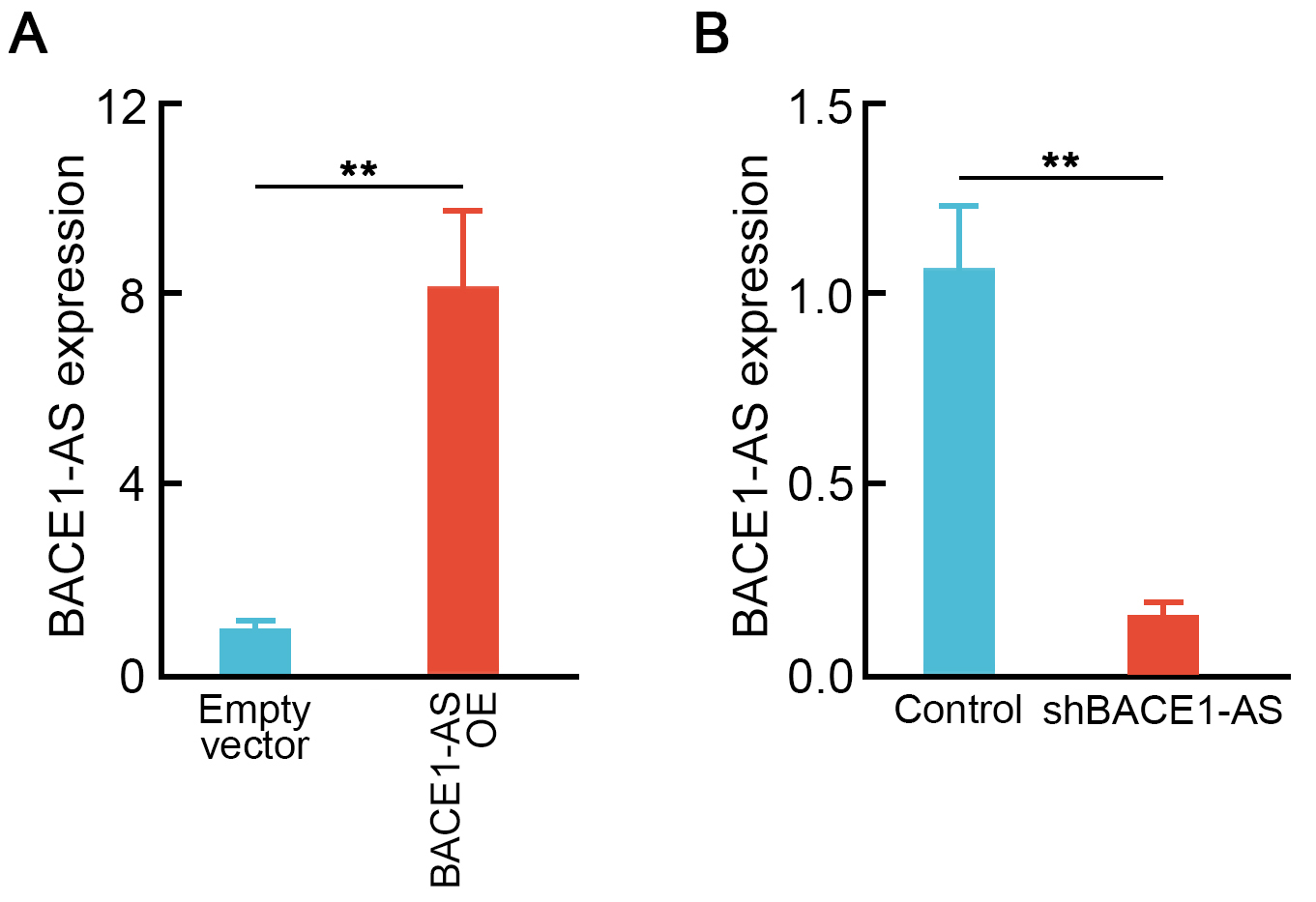

Supplement: Supplementary file 2 — Additional file 2: Figure S2. The efficiencies of BACE1-AS over-expression and knockdown in CRC cell lines. (A) BACE1-AS over-expression vector successfully up-regulated BACE1-AS level in HCT116 cells. (B) Transfection of BACE1-AS shRNA inhibited BACE1-AS expression in SW620 cells. [file 13046_2023_2881_MOESM2_ESM.jpg]

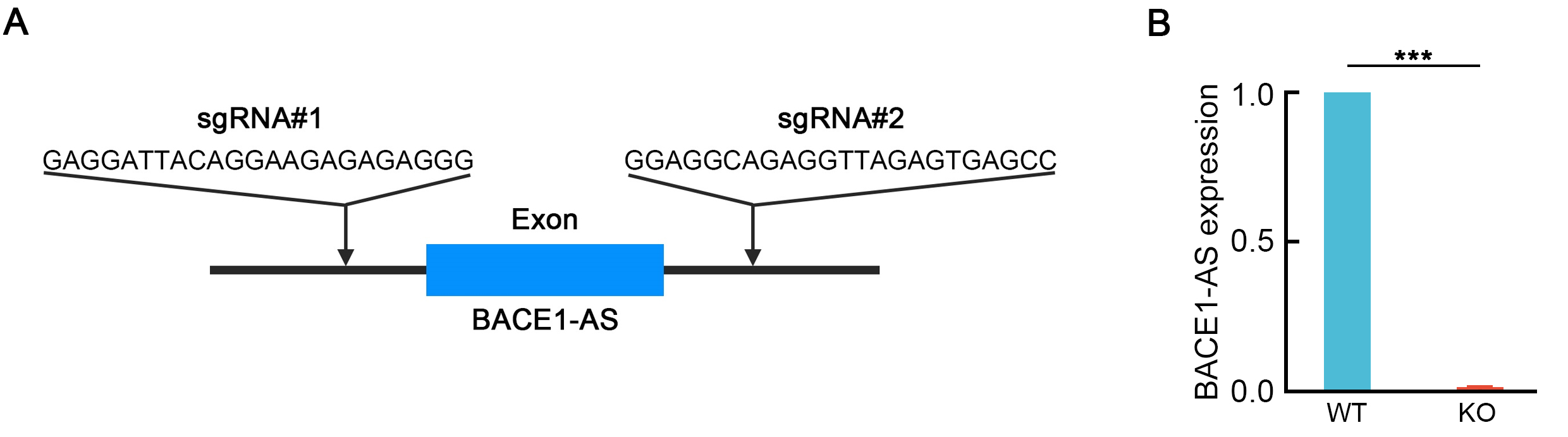

Supplement: Supplementary file 3 — Additional file 3: Figure S3. Schematic diagram of BACE1-AS knockout strategy. [file 13046_2023_2881_MOESM3_ESM.jpg]

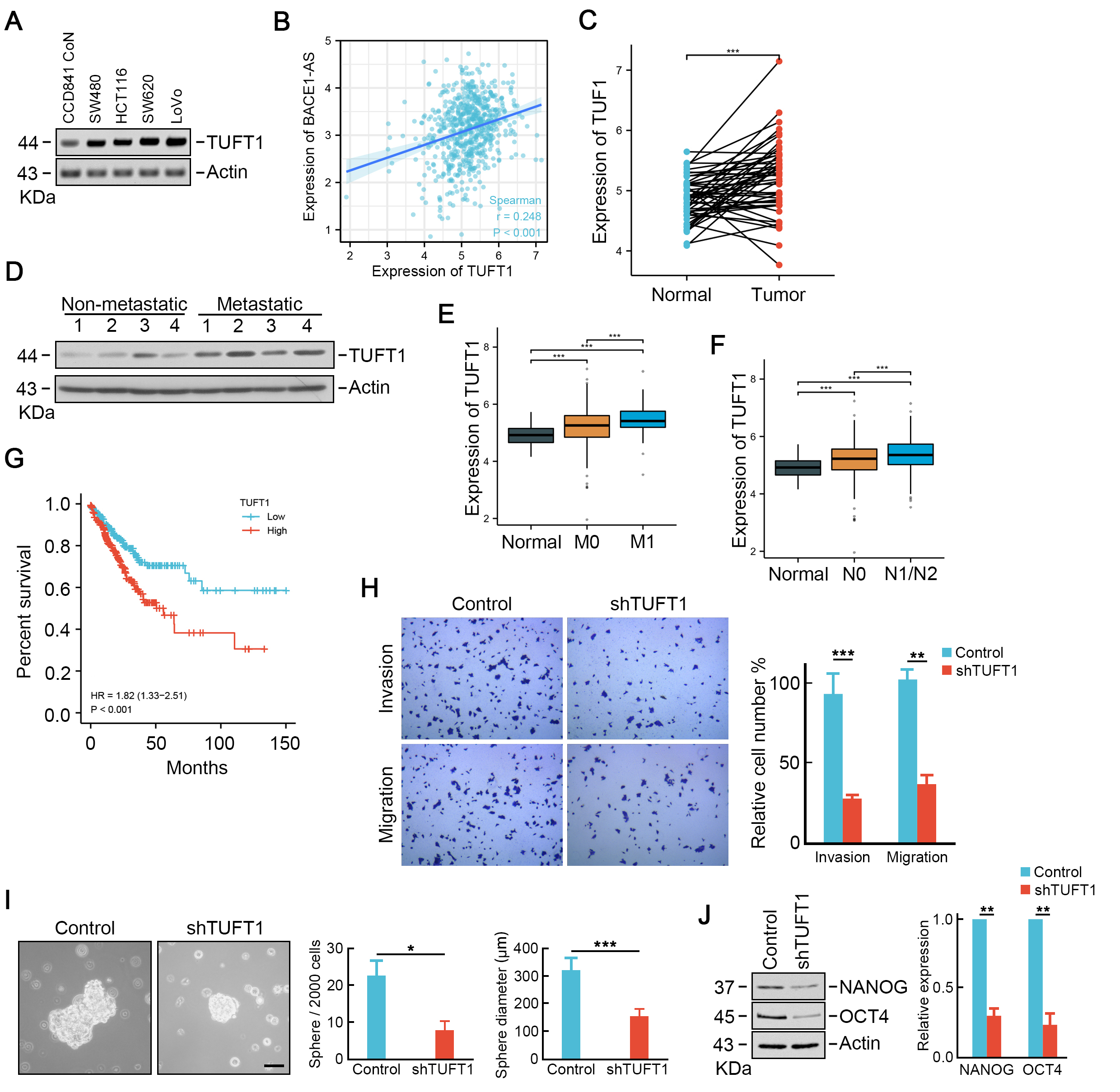

Supplement: Supplementary file 4 — Additional file 4: Figure S4. TUFT1 promotes CRC metastasis. (A) CRC cell lines expressed higher TUFT1 than CCD841 CoN normal human colonic epithelial cell line. (B) Positive correlation between BACE1-AS and TUFT1. Data from TCGA database. (C) Elevated TUFT1 was found in CRC tissues. Data from TCGA database. ***p<0.001. (D) TUFT1 levels in collected non-metastatic and metastatic CRC were determined by immunoblotting. (E-F) TUFT1 expression was increased in M stage (E) and N stage (F). M1 stage and N1/N2 stage tissues harbored even higher TUFT1 expression. ***p<0.001. (G) Higher TUFT1 level indicated worse CRC overall survival. (H) Knockdown of TUFT1 suppressed abilities of invasion and migration in SW620 cells. Representative images are shown (left panel). The bar graph shows the relative numbers of migration and invasion cells counted from three individual experiments (right panel). ***p<0.001. (I) Depletion of TUFT1 inhibited tumor sphere formation in SW620 cells. Scale bar = 100μm. *p<0.05, ***p<0.001. (J) Knockdown of TUFT1 suppressed the expressions of NANOG and OCT4 in SW620 cells. Actin served as a loading control. Representative blots from three experiments are shown (left panel). Ratios of levels of NANOG and OCT4 vs. Actin were calculated using NIH Image J 1.61 (right panel). **p<0.01. [file 13046_2023_2881_MOESM4_ESM.jpg]

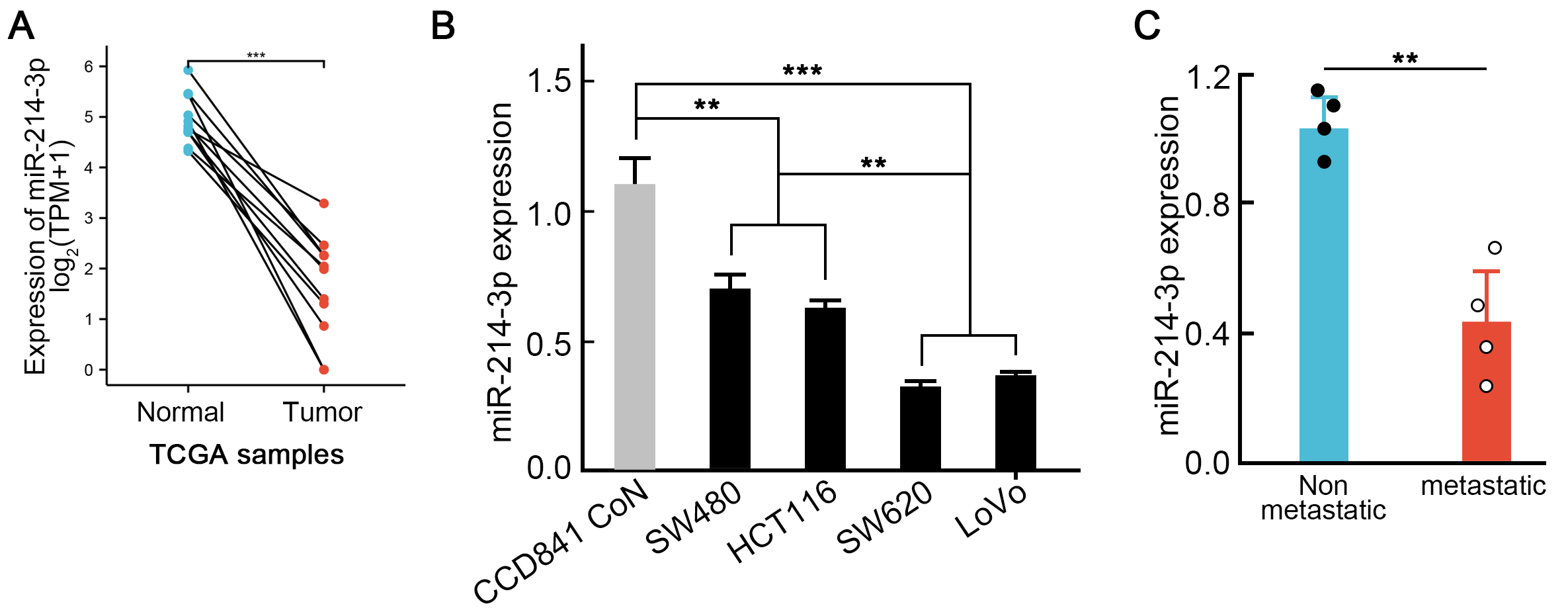

Supplement: Supplementary file 5 — Additional file 5: Figure S5. miR-214-3p is increased in metastatic CRC. (A) miR-214-3p was significantly decreased in CRC samples compared to normal tissues. Data from TCGA database. ***p<0.001. (B) miR-214-3p was significantly decreased in CRC cell lines compared to CCD841 CoN normal human colonic epithelial cell line. High metastasis potential cells (SW620 and LoVo) harbored even lower miR-214-3p than low metastasis potential cells (SW480 and HCT116). **p<0.01, ***p<0.001. (C) miR-214-3p expression in collected metastatic and non-metastatic CRC samples was examined by qRT-PCR. **p<0.01. [file 13046_2023_2881_MOESM5_ESM.jpg]
